# Financing Thresholds for Sustainability of Community Health Worker Programs for Patients Receiving Medicaid Across the United States

**DOI:** 10.1007/s10900-023-01290-w

**Published:** 2024-02-04

**Authors:** Sanjay Basu, Sadiq Y. Patel, Kiiera Robinson, Aaron Baum

**Affiliations:** 1 Clinical Product Development, Waymark Care, San Francisco, USA; 2 Department of Health Care Policy, Harvard Medical School, Boston, USA; 3 Icahn School of Medicine, Mount Sinai, New York, USA

**Keywords:** Community health workers, Alternative payment models, Medicaid

## Abstract

States have turned to novel Medicaid financing to pay for community health worker (CHW) programs, often through fee-for-service or capitated payments. We sought to estimate Medicaid payment rates to ensure CHW program sustainability. A microsimulation model was constructed to estimate CHW salaries, equipment, transportation, space, and benefits costs across the U.S. Fee-for-service rates per 30-min CHW visit (code 98960) and capitated rates were calculated for financial sustainability. The mean CHW hourly wage was $23.51, varying from $15.90 in Puerto Rico to $31.61 in Rhode Island. Overhead per work hour averaged $43.65 nationwide, and was highest for transportation among other overhead categories (65.1% of overhead). The minimum fee-for-service rate for a 30-min visit was $53.24 (95% CI $24.80, $91.11), varying from $40.44 in South Dakota to $70.89 in Washington D.C. The minimum capitated rate was $140.18 per member per month (95% CI $105.94, $260.90), varying from $113.55 in South Dakota to $176.58 in Washington D.C. Rates varied minimally by metro status but more by panel size. Higher Medicaid fee-for-service and capitated rates than currently used may be needed to support financial viability of CHW programs. A revised payment estimation approach may help state officials, health systems and plans discussing CHW program sustainability.

## Introduction

Community health worker (CHW) programs have demonstrated improvements in health outcomes, especially for low-income or otherwise marginalized populations who face significant barriers to receiving quality healthcare services [1–3]. CHWs are generally people from the same community as a patient, who provide critical social and emotional support, health education, and/or navigation assistance that supplements the work of other healthcare providers to assist in improving access to or quality of healthcare services for a patient [4]. CHW program evaluations have included randomized controlled trials among diverse populations, showing improvements of health outcomes and healthcare costs, particularly among Medicaid beneficiaries [5, 6].

CHW programs have faced considerable financing challenges despite the evidence of their benefits to health outcomes and healthcare costs. Many CHW programs have been financed under temporary grants, which has posed challenges for their long-term viability [7]. Recently, CHW programs have been included among states’ Medicaid 1115 waivers and related demonstration programs that aim to direct Medicaid dollars to CHW services [8]. CHW programs funded by Medicaid financing are either paid through fee-for-service (FFS) payments for visits between CHWs and patients, or capitated per-member-per-month (PMPM) payments to CHWs caring for a population panel of patients as part of a Medicaid managed care organization or a healthcare practice that has entered into value-based contracting. What remains unclear is how much financing should be allotted in FFS or PMPM payments to enable CHW programs to be financially sustainable. CHW programs, unlike standard medical care in clinics or hospitals, often require considerable transportation time, mobile equipment, and associated expenses, alongside flexible systems for supervision and support to ensure safety of CHWs and effectiveness in delivering roving, within-community services.

In the current study, we seek to establish and apply a rigorous approach to estimating the levels of Medicaid FFS or PMPM payments necessary to support the financial viability of CHW programs across all 50 US states, Washington D.C. and Puerto Rico. We develop and implement a microsimulation model that synthesizes data on CHW salaries, supervision, equipment, transportation, benefits and related costs across the US. We use the model to estimate the levels of financing needed to cover CHW program costs across different geographies, while maintaining patient volume or panel sizes that are consistent with the randomized trials showing benefits of CHWs on patient outcomes and healthcare costs.

## Methods

### Methods Overview

**Fig. 1:**
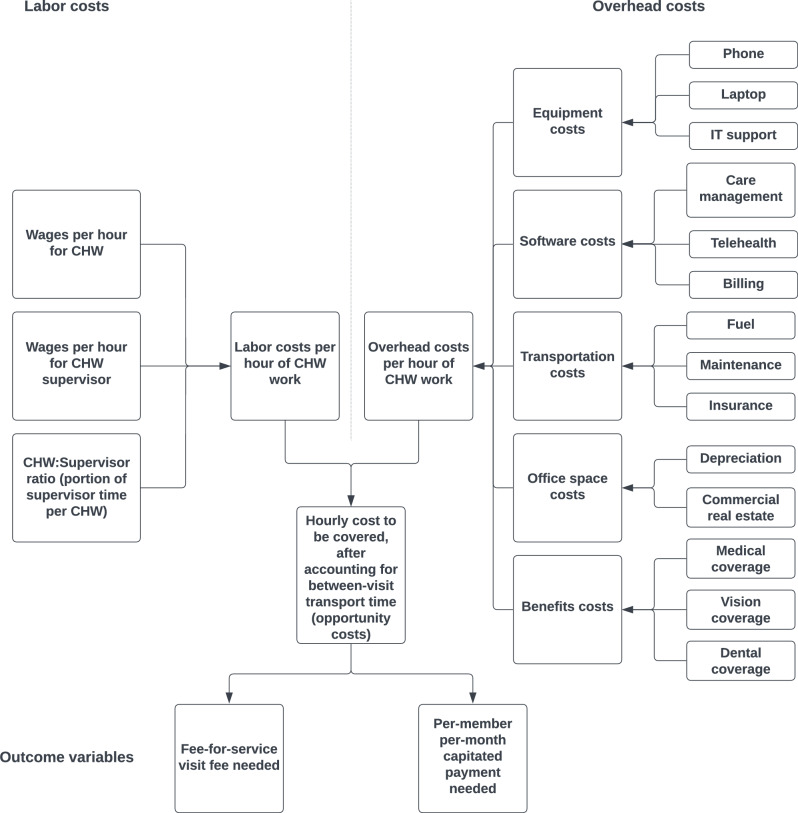
Microsimulation model diagram. The diagram depicts how input variables were synthesized into an estimate of the outcome variables

We constructed a microsimulation model (Fig. 1), which is a type of simulation that accounts for the covariation among input variables to estimate variations in output variables across locations (e.g., capturing how wages or transportation costs may be higher, and therefore subsequent payments may need to be higher in a given location). In our prespecified economic analysis plan (see “Data Availability”), we chose microsimulation because wages and costs related to CHW programs are expected to be highly variable across geographies, and hence we judged that capturing variations through a microsimulation, rather than only using national average estimates for cost, would be important to ensure the financial viability of CHW programs that are typically susceptible to local variations in labor and overhead expenditure. Our methods and reporting corresponded to the Consolidated Health Economic Evaluation Reporting Standards (see Appendix for checklist) [9].

### Input Variables and Data Sources

Input variables and data sources for the model are summarized in Table 1, and further detailed in the Appendix by state (including Washington D.C. and Puerto Rico) and by metropolitan or non-metropolitan statistical area within states. The study population was defined as US-based CHW programs serving Medicaid patients.

**Table 1:** Input variables and data sources for the model

Variables	Source(s)
*Wages ($ per hour)*	
Community health workers (CHWs)	Bureau of Labor Statistics Occupational Employment and Wage Statistics (OEWS) database [10]
CHW supervisors	Bureau of Labor Statistics Occupational Employment and Wage Statistics (OEWS) database [10]
*Overhead expenditures ($ per CHW hour worked)*	
Equipment: Mobile phone plan, laptop, IT support	Hardware and service surveys [13–15]
Software: Care management records, panel management, telehealth, billing systems	Software survey [16]
Transportation: Fuel costs, maintenance, insurance and depreciation	Automotive surveys [18, 19]
Office space	Commercial real estate database [20]
Benefits	Employer databases [21, 22]

Values across states and sub-state metropolitan and non-metropolitan areas are provided in the Appendix

The first set of input variables were labor costs (Fig. 1). CHW and CHW supervisor wages from the Bureau of Labor Statistics Occupational Employment and Wage Statistics (OEWS) database, which provides wage estimates from 82 million people (62% of total national employment) working at 1.1 million non-farm employer establishments surveyed through six semiannual panels collected over a 3-year period (2018–2021) [10]. In the main analysis, we used CHW supervisor wages for Registered Nurse (RN) care managers, to correspond to a common supervision structure reported in systematic reviews, accounting for a typical 1:8 ratio of RN supervisors to CHWs; in a sensitivity analysis (detailed further below), we varied the CHW supervisor to correspond to a Master’s-level Medical Social Worker (MSW) with a 1:6 ratio of MSW to CHW, to address another commonly-reported supervisor profile [11, 12]. Both the mean and standard deviations around the mean for CHW and CHW supervisor salaries were obtained from the OEWS database, for each state and each metropolitan or non-metropolitan statistical area within states (see Appendix).

The second set of input variables were overhead expenditures (Fig. 1). Based on prior accounting efforts to estimate costs among CHW programs, the overhead expenditure categories we considered in the model included equipment, software, transportation, space, and benefits. For equipment, we gathered the cost of annual mobile phone plan with unrestricted text, call and data including mobile Wi-Fi hotspot; the annualized cost of a Chromebook laptop over a typical four-year lifecycle; and costs of ongoing Information Technology (IT) support [13–15]. For software, we incorporated costs of CHW and CHW supervisor accounts on the most popular patient care management software systems incorporating records management, panel management, telehealth functionalities and billing [16]. For transportation, we gathered the typical time and associated mileage and gasoline expenses required for transportation from a CHW home to a patient home or CHW workspace, based on commute times by location [17]. The transportation time was included to account for both the costs of transportation itself (fuel costs, maintenance, insurance and depreciation) and opportunity costs (the wage costs to support CHWs for their time in transit, which would need to be indirectly compensated by visit-based FFS or panel-based PMPM payments) [18, 19]. For space, we computed the average cost of office space per person in the locale from a national real estate database [20]. For benefits, we assessed the overhead costs of initial and continuing CHW education based on a review of state education requirements associated with CHW certification, along with provision and administration of employer-sponsored healthcare insurance coverage (medical, dental, and vision) with a health maintenance organization or preferred-provider organization plan based on the average benefit expenditure rate reported among healthcare organizations in a national employer-sponsored benefits survey [21, 22].

### Outcome Definitions

We calculated two outcomes, estimating the threshold value for each outcome to cover the total annual expenses for a CHW, after incorporating the above costs. The two outcomes were: (i) the Medicaid FFS payment rate per a 30-min visit between a CHW and an individual patient (typically Medicaid billing code 98960, which most states use as the basis for CHW payment [21]), either in person or virtually (telephone or video); or (ii) the Medicaid PMPM payment rate for every patient on the CHW’s panel at a given time.

For both outcomes, we computed the payment rates for a full-time CHW with 2080 h of work per year, with a mean patient panel of 65 patients at a given moment, and a mean 6-month duration of service per patient (varied in sensitivity analyses detailed below) [12]. All costs were summed from a CHW service provider perspective (accounting for labor and overhead including opportunity cost) over a one-year time horizon, and updated to undiscounted 2023 US Dollars using the Consumer Price Index to account for inflation [23].

### Computation Methods

We estimated each outcome separately, assuming each CHW would be paid under either a Medicaid FFS or capitated PMPM contract. For each outcome, we first computed the total monthly cost to support each CHW each month by summing the total costs listed above and detailed in Table 1, scaling the estimate to a cost per CHW per month including overhead costs associated with that CHW. We then estimated the time left for visits and the associated transportation time to estimate the typical visit rate per CHW per month, with a ratio of 1:3 of in-person to virtual (phone or video) visits [24]. Finally, we estimated each of the outcome variables by estimating what threshold for payment would be the minimum amount to meet the total monthly costs given the visit rate or typical patient panel size per CHW (65 patients per CHW, each enrolled for 6 months) [12]. We completed the calculations for each state and for each sub-state metropolitan or non-metropolitan area. For validation, we compared our estimates to the program costs available from CHW program costs in two states (Virginia and Washington state) for which independent and complete wage and overhead expenditure data were available [25].

### Sensitivity and Uncertainty Analysis

For each outcome, we sampled 10,000 times with replacement from normal distributions constructed around the mean and standard deviation of each input variable (Appendix) to estimate the mean and 95% credible interval (CI) around each outcome estimate [26].

We performed two sensitivity analyses. In the first sensitivity analysis, we swapped out the salary and overhead expenditures for an RN care manager supervisor for each team of eight CHWs for the salary and overhead expenditures for an MSW supervisor for each team of six CHWs [11, 12]. In the second sensitivity analysis, we varied the size of the patient panel associated with each CHW from a low of 15 to a high of 100 to understand the implications of widely varying panel size among CHW programs.

## Results

### Descriptive Statistics on the Input Variables

**Fig. 2:**
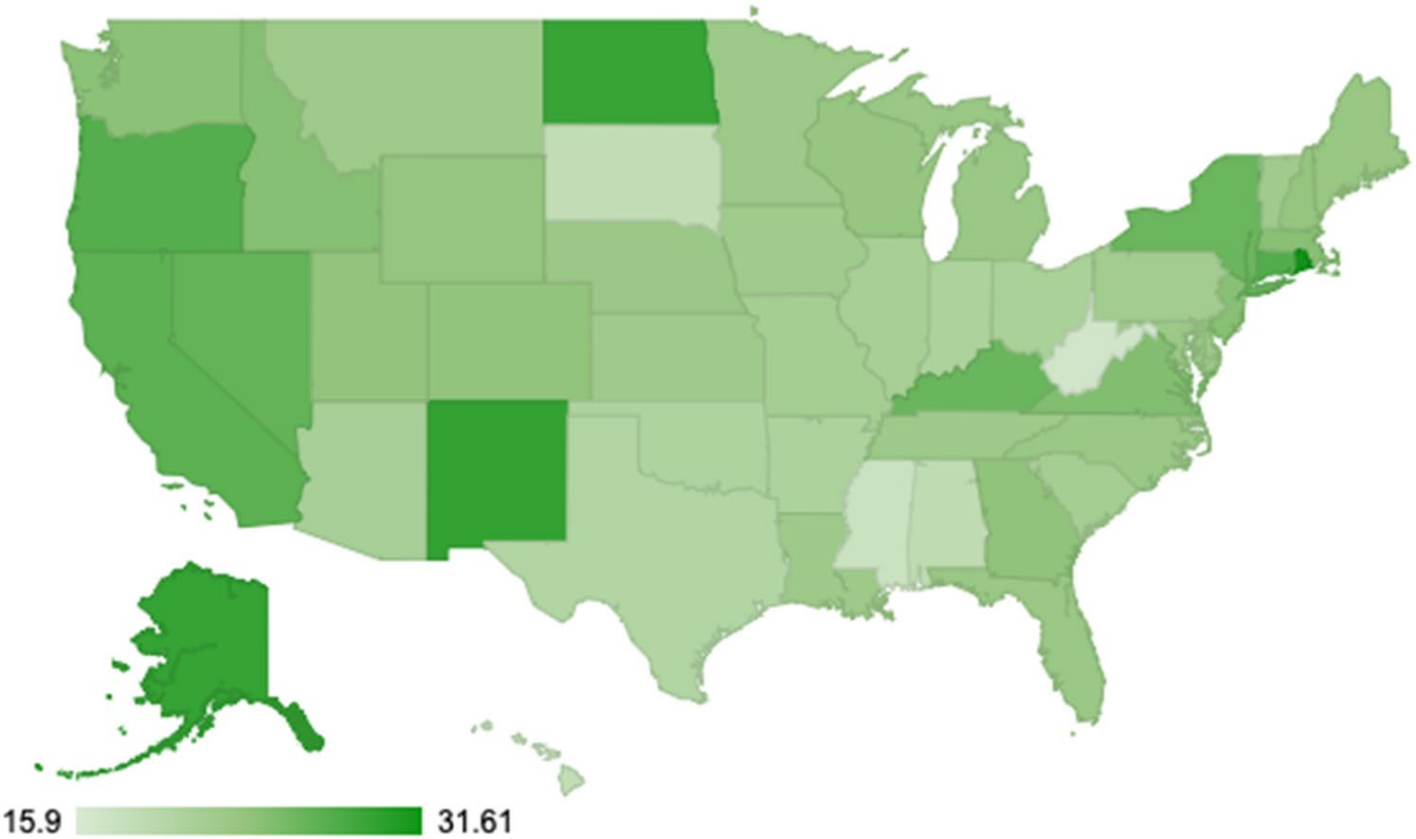
State variations in wages among community health workers (CHW). Wages are expressed in dollars per hour of work in 2023 US Dollars using the Consumer Price Index to account for inflation [23]

CHW wages estimated in the Bureau of Labor Statistics OEWS database had a mean national hourly wage of $23.51 (varying from $15.90 in Puerto Rico to $31.61 in Rhode Island; Fig. 2), with wages averaging $22.54 in metropolitan areas and $21.13 in non-metro areas. CHW supervisor wages when including RN care managers as supervisors had a mean national hourly wage of $39.88 (varying from $17.96 in Puerto Rico to $64.10 in California, and averaging $40.87 in metro and $38.93 in non-metro areas), where MSW supervisors had a mean national hourly wage of $29.37 (varying from $16.22 in Puerto Rico to $42.49 in California; Fig. 2).

Overhead expenditures (equipment, software, transportation, space, and benefits) per CHW work hour averaged $43.65 nationwide (varying from $19.21 in North Dakota to $196.41 in New York, and averaging $43.84 in metro and $43.46 in non-metro areas). Among the varied overhead expenditures, the highest sub-cost was universally for transportation, which constituted a mean of 65.1% of the overhead expenditure nationally (varying from 21.0% in Hawaii to 94.2% in Arkansas). In our face validity checks, wage and overhead expenditure data did not differ between model estimates and independent program data available from two states (Appendix).

### Fee-for-Service Payment Rate Minimums

**Fig. 3:**
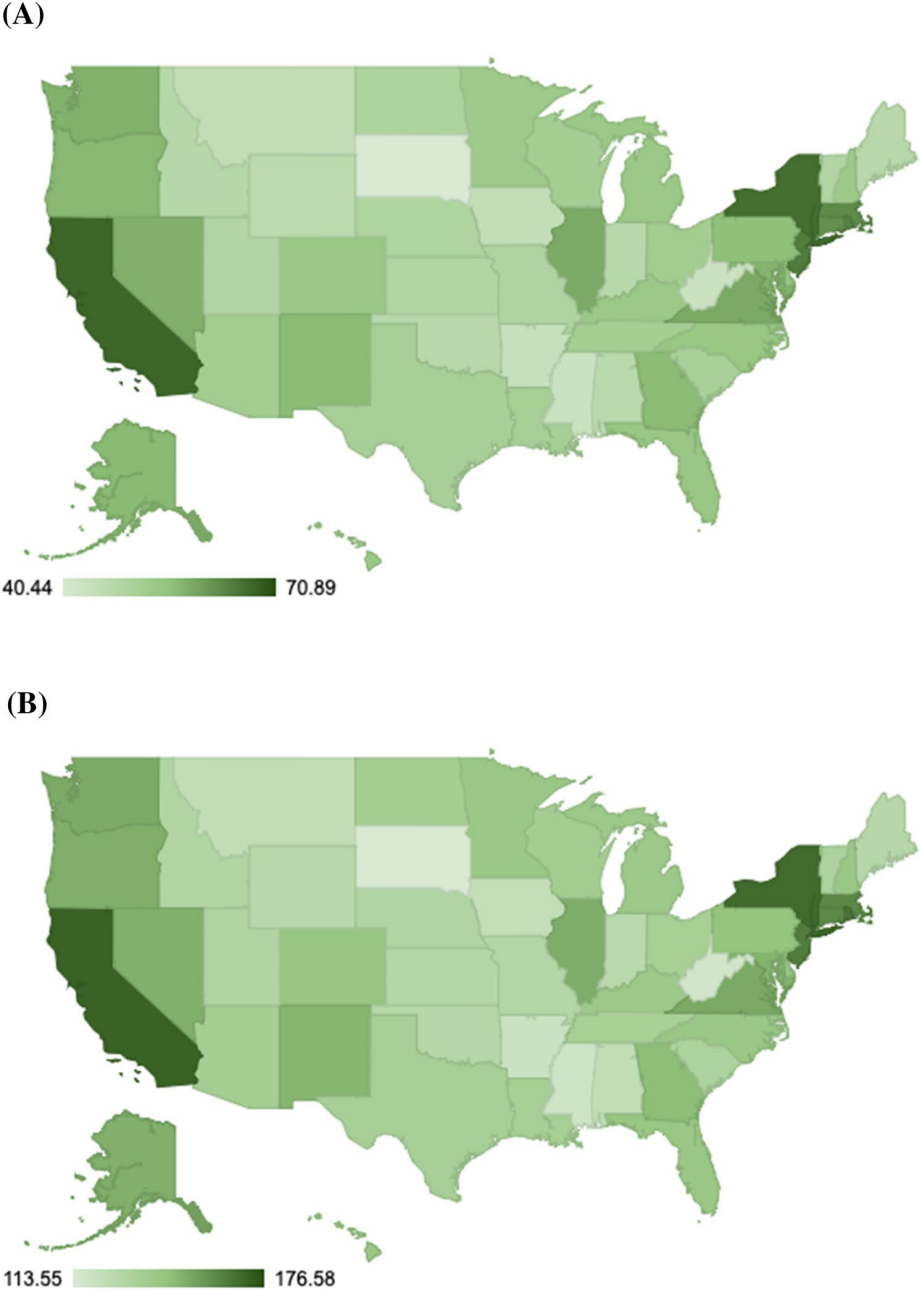
State variations in the payment threshold rates for sustaining community health worker (CHW) programs. The **A** minimum fee-for-service payment rate was computed for a 30-min CHW visit (Medicaid billing code 98960), and the **B** minimum capitated payment rate per member per month (PMPM), after considering the wages and overhead costs for CHW programs

The minimum Medicaid fee-for-service payment rate for a 30-min CHW visit (Medicaid billing code 98960), after considering the wages and overhead costs for CHWs, was estimated to be a mean of $53.24 nationwide (95% CI $24.80, $91.11). Among states, the minimum varied from $40.44 in South Dakota (95% CI $17.65, $71.09) to $70.89 in Washington D.C. (95% CI $32.08, $120.25) due to the combination of wages and overhead costs (Table 2, Fig. 3).

**Table 2:** Estimated minimum payment threshold rates for sustaining community health worker (CHW) programs across states

State	Minimum fee-for-service (FFS) payment per visit	95% CI (FFS lower)	95% CI (FFS upper)	Minimum capitated payment per month per patient (PMPM)	95% CI (PMPM lower)	95% CI (PMPM upper)
Alabama	$47.10	$25.03	$76.71	$124.46	$89.01	$243.99
Alaska	$56.89	$22.69	$101.91	$150.42	$119.37	$266.72
Arizona	$52.01	$25.36	$87.17	$136.57	$101.81	$254.57
Arkansas	$44.54	$21.46	$75.29	$120.02	$88.02	$240.32
California	$67.81	$31.48	$116.74	$171.18	$132.83	$298.64
Colorado	$54.11	$23.94	$95.09	$143.07	$109.62	$263.14
Connecticut	$62.32	$28.17	$107.07	$160.88	$125.20	$280.90
Delaware	$54.69	$25.24	$93.43	$142.64	$107.90	$266.56
District of Columbia	$70.89	$32.08	$120.25	$176.58	$139.26	$305.13
Florida	$54.45	$26.90	$92.16	$142.10	$106.20	$260.65
Georgia	$56.70	$27.91	$95.00	$146.60	$109.73	$273.31
Hawaii	$53.39	$23.49	$93.45	$142.18	$109.05	$259.47
Idaho	$47.97	$20.94	$83.96	$131.26	$99.81	$247.98
Illinois	$58.86	$29.34	$98.39	$150.82	$112.03	$277.58
Indiana	$47.35	$22.53	$80.80	$127.53	$94.35	$247.32
Iowa	$45.41	$20.89	$78.43	$123.64	$92.25	$238.82
Kansas	$48.95	$22.94	$83.56	$129.69	$96.86	$245.18
Kentucky	$53.83	$25.50	$91.70	$140.56	$105.38	$265.86
Louisiana	$51.32	$25.29	$86.67	$135.90	$100.74	$255.86
Maine	$47.69	$20.29	$83.77	$129.08	$98.44	$247.03
Maryland	$57.63	$27.61	$98.30	$149.12	$112.04	$276.00
Massachusetts	$63.08	$28.99	$108.80	$161.01	$123.86	$284.78
Michigan	$53.81	$24.76	$92.05	$140.73	$106.48	$260.81
Minnesota	$53.18	$23.98	$91.93	$141.90	$107.35	$268.07
Mississippi	$43.70	$22.27	$71.94	$118.29	$85.00	$236.25
Missouri	$49.46	$24.00	$83.10	$130.14	$96.80	$246.72
Montana	$45.53	$19.24	$79.71	$124.13	$95.60	$236.01
Nebraska	$49.45	$23.07	$83.49	$130.89	$98.46	$248.70
Nevada	$58.26	$26.68	$100.24	$150.26	$115.59	$272.10
New Hampshire	$53.46	$24.86	$92.85	$142.16	$107.42	$260.36
New Jersey	$64.83	$31.39	$109.37	$163.94	$124.95	$289.53
New Mexico	$56.70	$25.41	$98.44	$148.81	$114.68	$271.06
New York	$66.76	$31.27	$114.03	$169.20	$131.07	$295.57
North Carolina	$54.52	$26.74	$92.28	$141.77	$105.66	$263.16
North Dakota	$49.83	$19.22	$89.83	$136.74	$107.44	$254.37
Ohio	$51.94	$25.15	$86.98	$136.22	$101.42	$258.94
Oklahoma	$47.58	$22.87	$80.73	$129.19	$94.72	$253.22
Oregon	$57.22	$24.94	$99.47	$150.65	$116.53	$274.67
Pennsylvania	$56.09	$27.16	$94.33	$144.57	$108.70	$261.63
Puerto Rico	$44.60	$25.01	$70.68	$118.64	$81.61	$244.01
Rhode Island	$64.02	$28.30	$111.88	$166.31	$129.51	$291.78
South Carolina	$50.85	$25.03	$85.23	$133.70	$98.71	$255.96
South Dakota	$40.44	$17.65	$71.09	$113.55	$84.58	$229.03
Tennessee	$51.60	$25.16	$86.50	$135.36	$100.81	$256.67
Texas	$51.11	$25.14	$86.68	$135.54	$99.98	$260.18
Utah	$48.66	$21.30	$85.20	$131.51	$100.58	$243.34
Vermont	$48.96	$21.13	$85.98	$133.04	$101.90	$250.74
Virginia	$59.05	$28.36	$100.15	$152.11	$115.71	$273.18
Washington	$58.04	$26.16	$101.28	$151.74	$116.46	$275.71
West Virginia	$43.54	$21.80	$72.65	$117.24	$84.77	$235.24
Wisconsin	$51.51	$23.47	$88.66	$137.57	$104.58	$256.70
Wyoming	$46.91	$19.81	$82.53	$128.28	$98.28	$243.05

The minimum fee-for-service (FFS) payment rate was computed for a 30-min CHW visit (Medicaid billing code 98960), and the minimum capitated payment rate per member per month (PMPM), after considering the wages and overhead costs for CHW programs

Among sub-state metropolitan or non-metropolitan areas, the minimum fee-for-service payment rate for a 30-min CHW visit varied from $40.24 in the Las Cruces, New Mexico area (95% CI $17.60, $69.89) to $78.08 in the Napa, California area (95% CI $33.85, $136.93; see Appendix). Fee-for-service minimums were typically higher in metropolitan than in non-metropolitan areas but with overlapping credible intervals ($52.38 in metro, 95% CI $24.34, $89.77; $49.82 in non-metro, 95% CI $23.29, $81.15).

### Capitated Payment Rate Minimums

The minimum Medicaid capitated payment rate per member per month (PMPM), after considering the same overhead costs as above, was estimated to be a mean of $140.18 nationwide (95% CI $105.94, $260.90). Among states, the minimum varied from $113.55 in South Dakota (95% CI $84.58, $229.03) to $176.58 in Washington D.C. (95% CI $132.83, $298.64; Table 2, Fig. 3).

Among sub-state metropolitan or non-metropolitan areas, the minimum PMPM rate varied from $111.76 in the Las Cruces, New Mexico area (95% CI $83.37, $223.62) to $196.08 in the Napa, California area (95% CI $156.88, $326.14). Capitated minimums were typically higher in metropolitan than in non-metropolitan areas but with overlapping credible intervals ($138.41 in metro, 95% CI $104.51, $258.02; $132.78 in non-metro, 95% CI $99.54, $250.80).

### Sensitivity Analyses

In the first sensitivity analysis, when we swapped out the expenditures for an RN care manager supervisor (1 for each 8 CHWs) for an MSW supervisor (1 for each six CHWs), the minimum fee-for-service payment rate for a 30-min CHW visit was estimated as a mean of $52.80 nationwide (95% CI $24.78, $90.08), while the minimum capitated PMPM rate was estimated as a mean of $139.02 nationwide (95% CI $104.87, $258.13; see Appendix for data across states).

In the second sensitivity analysis, we varied the size of the patient panel associated with each CHW from a low of 15 to a high of 100. At the lowest panel size of 15, the minimum fee-for-service payment rate rose to a mean of $230.72 (95% CI $107.45, $394.83), and capitated PMPM to a mean of $607.45 (95% CI $459.09, $1130.55; see Appendix). At the highest panel size of 100, the minimum fee-for-service payment rate declined to a mean of $34.61 (95% CI $16.12, $59.22) and capitated PMPM to a mean of $91.12 (95%CI $68.88, $169.58; see Appendix).

## Discussion

As state Medicaid agencies seek to provide sustainable funding for CHW programs, we established and applied a rigorous approach to estimating the levels of Medicaid FFS or PMPM payments to support the financial viability of CHW programs across all 50 US states, Washington D.C. and Puerto Rico. In the past, payments for services outside of face-to-face medical encounters in hospitals or clinic settings have lacked a systematic approach to establishing their basis, and CHW programs particularly face numerous challenges that differ from traditional medical visits—such as potentially meaningful transportation time to patient locations.

We found that the threshold minimum levels of both Medicaid FFS and PMPM payments estimated through our microsimulation model to sustain CHW programs were typically much higher than those currently publicly disclosed by Medicaid officials. For example, whereas the estimates in our main analysis indicated a minimum FFS rate of $53.24 nationally for a 30-min Medicaid billing code 98960, publicly-accessible Medicaid state plan amendments and waivers lack clarity on how their rates were established and indicate variations from $9.60 in Indiana to $30.89 in South Dakota [27, 28]—all much lower than the threshold to achieve financial sustainability, per our estimates. Similarly, while our minimum capitated rate estimate was $140.18 PMPM, capitated rates that are publicly disclosed appear lower, on the order of $1.65 in Maine and $9.65 in Idaho [29, 30], though such data are not systematically publicly disclosed per recent reviews of the subject [31]. Additionally, our findings indicate that meaningful accounting of overhead expenditure and local wage rates is important to account for when computing the funding levels necessary to sustain a CHW program. Overall overhead expenditures did not differ markedly between metro and non-metro areas, as transport time was higher in non-metro areas but was counterbalanced by higher employee benefit and space costs in metro areas.

As with any study based on simulations, our study is subject to limitations associated with model assumptions. First, we considered two common types of supervisor types for CHWs, but did not consider all possibilities; CHW programs vary widely in their regulation and implementation. Secondly, we consciously focused on a suite of expenditures including standard employee benefits at the overhead rates seen in the healthcare sector generally, although some CHW programs do not offer such employee benefits to CHWs. We chose to include benefit overhead expenditures intentionally, as we did not want to compute minimum thresholds for payment that did not consider the need for sustainable, long-term career potential for CHWs, and particularly wanted to ensure consideration of providing healthcare coverage to employees who themselves are providing healthcare services. Third, our study revealed that the threshold estimates for Medicaid fee-for-service and capitated rates could be sensitive to the panel size adopted by CHWs; while we adopted panel sizes from published research articles reporting successful CHW programs, it is possible that higher and lower panel sizes may be successful among varied patient populations, and associated payments would need to be adjusted accordingly. Finally, while our study focused on patients receiving Medicaid, for whom CHW programs have been carefully studied, CHW services are increasingly available to uninsured, Medicare, and commercially-insured patients who have a different burden of illness and potentially different cost structure for their infrastructure.

We believe future research on the subject of CHW payment would benefit from the broader disclosure of payment levels, as publicly-available listings for CHW payment rates were limited to a few states with Medicaid state plan amendments or waivers requiring such disclosure. Additionally, further study may be required into the intangible opportunity costs facing CHWs and affecting program sustainability, such as the availability and affordability of childcare, and of tools and data technologies needed to ensure safety when performing community-based home visits.

While such opportunities for further research may be present in coming years, our current study synthesized available data on CHW salaries, supervision, equipment, transportation, benefits and related costs across the US to provide estimates of the levels of financing needed to cover CHW program costs across different geographies. We found that to maintain patient volume or panel sizes that are consistent with the randomized trials showing benefits of CHWs on patient outcomes and healthcare costs, levels of fee-for-service and capitated program payment for CHW programs would likely need to be considerably higher than current publicly-available data sources suggest are offered for Medicaid-covered CHW services.

## Figures and Tables

**Fig. 4:**
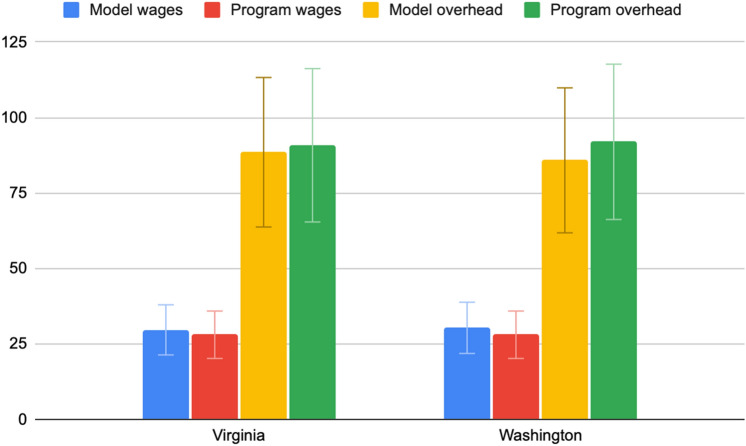
Validation of program cost estimates per CHW per hour (2023 $US), versus independent data from CHW programs in two states (*Waymark Evidence/Clinical Trials*. University of California San Francisco; 2023. Accessed May 16, 2023. https://digital.health/product-detail/waymark)

## Data Availability

All data used for this study are publicly available, and statistical/modeling code are also available at https://github.com/sanjaybasu/chw_payment.

## Appendix

See Tables 3, 4, 5, 6, 7, and 8 and Fig. 4.

**Table 3:** Wage data for CHWs and CHW supervisors (RN care coordinators and Master’s-level Medical Social Worker) by state

State	CHW wage, $/hr (SD)	RN wage, $/hr (SD)	MSW wage, $/hr (SD)
Alabama	$18.88 (2.31)	$32.17 (8.44)	$24.78 (6.80)
Alaska	$29.38 (7.56)	$49.67 (13.25)	$32.9 (9.22)
Arizona	$21.39 (4.79)	$41.7 (7.42)	$30.35 (9.20)
Arkansas	$20.8 (3.53)	$31.98 (6.62)	$27.23 (5.04)
California	$27.07 (9.33)	$64.1 (20.37)	$42.49 (14.71)
Colorado	$23.84 (5.74)	$41.63 (7.86)	$28.04 (9.21)
Connecticut	$27.61 (8.01)	$45.32 (7.77)	$35.44 (7.03)
Delaware	$22.78 (8.42)	$40.88 (6.99)	$27.93 (11.15)
District of Columbia	$30.89 (4.90)	$47.23 (9.81)	$30.22 (7.55)
Florida	$22.94 (8.97)	$38.42 (6.68)	$26.57 (6.80)
Georgia	$23.88 (7.98)	$40.95 (11.22)	$27.71 (8.14)
Hawaii	$18.47 (3.36)	$54.43 (13.79)	$23.37 (5.52)
Idaho	$24.54 (5.81)	$37.79 (6.19)	$34.93 (9.39)
Illinois	$21.44 (4.58)	$39.53 (9.08)	$29.62 (8.24)
Indiana	$20.89 (5.38)	$36.34 (6.63)	$28.38 (9.61)
Iowa	$22.26 (7.32)	$33.35 (5.69)	$27.47 (6.33)
Kansas	$22.43 (4.08)	$34.61 (6.47)	$27.85 (4.69)
Kentucky	$26.21 (3.62)	$37.32 (6.44)	$29.01 (8.34)
Louisiana	$22.54 (6.52)	$36.5 (6.69)	$27.06 (7.07)
Maine	$23.17 (6.68)	$37.22 (6.58)	$28.76 (5.66)
Maryland	$22.75 (7.54)	$42.3 (8.45)	$29.05 (4.71)
Massachusetts	$24.37 (6.25)	$50.07 (17.68)	$28.36 (10.64)
Michigan	$22.93 (4.61)	$38.78 (7.60)	$31.46 (10.11)
Minnesota	$22.7 (4.68)	$42.72 (9.42)	$28.55 (6.25)
Mississippi	$17.62 (5.20)	$32.66 (6.85)	$30.81 (7.67)
Missouri	$21.75 (6.09)	$34.55 (7.02)	$26.33 (7.46)
Montana	$22.47 (5.63)	$37.67 (6.67)	$24.8 (8.49)
Nebraska	$22.86 (9.05)	$35.34 (5.81)	$27.56 (6.12)
Nevada	$26.6 (4.51)	$46.3 (8.51)	$26.12 (6.42)
New Hampshire	$23.58 (6.97)	$40.11 (9.54)	$33.63 (9.29)
New Jersey	$24.41 (4.94)	$46.48 (8.18)	$34.23 (6.55)
New Mexico	$29.53 (10.23)	$41.15 (7.39)	$34.45 (6.89)
New York	$26.13 (7.97)	$48.14 (9.64)	$30.38 (8.75)
North Carolina	$22.92 (5.48)	$37.22 (6.95)	$29.06 (10.30)
North Dakota	$29.27 (12.97)	$36.06 (6.37)	$30.34 (7.46)
Ohio	$21.17 (5.34)	$37.72 (5.95)	$28.31 (4.78)
Oklahoma	$20.68 (5.92)	$36.98 (6.58)	$28.71 (6.88)
Oregon	$27.46 (10.49)	$51.26 (9.1)	$25.49 (7.74)
Pennsylvania	$22.01 (4.63)	$38.76 (9.6)	$37.09 (8.53)
Puerto Rico	$15.9 (5.47)	$17.96 (3.19)	$30.01 (8.38)
Rhode Island	$31.61 (15.11)	$42.43 (9.07)	$16.22 (4.27)
South Carolina	$21.73 (5.74)	$35.74 (6.76)	$35.63 (7.09)
South Dakota	$18.52 (4.68)	$31.01 (7.54)	$28.5 (8.29)
Tennessee	$22.54 (4.52)	$34.85 (6.39)	$23.54 (6.55)
Texas	$20.3 (3.77)	$40.54 (10.49)	$25.63 (7.85)
Utah	$23.75 (7.33)	$36.73 (6.62)	$30.05 (9.11)
Vermont	$22.3 (5.74)	$38.46 (8.38)	$32.72 (7.52)
Virginia	$24.70 (7.05)	$39.36 (9.86)	$31.39 (10.39)
Washington	$24.18 (7.01)	$48.88 (12.76)	$27.68 (9.30)
West Virginia	$16.91 (5.23)	$34.73 (6.91)	$34.27 (10.42)
Wisconsin	$23.31 (6.23)	$38.94 (6.81)	$27.37 (5.67)
Wyoming	$23.51 (8.00)	$38.95 (6.85)	$29.38 (6.98)

Additional input data across the domains of equipment, software, transportation, space, and benefits can be downloaded in spreadsheet format from: https://github.com/sanjaybasu/chw_payment

**Table 4:** Wage data for CHWs and CHW supervisors (RN care coordinators and Master’s-level Medical Social Worker) by metropolitan and non-metropolitan areas

Area (census statistical area code number)	CHW wage, $/hr (SD)	RN wage, $/hr (SD)	MSW wage, $/hr (SD)
Abilene, TX (0010180)	$19.31 (2.56)	$34.34 (1.5)	$40.54 (4.92)
Akron, OH (0010420)	$22.86 (5.86)	$37.27 (5.63)	$37.72 (6.33)
Alaska nonmetropolitan area (0200006)	$32.29 (8.47)	$50.17 (14.11)	$49.67 (6.42)
Albany-Schenectady-Troy, NY (0010580)	$29.62 (9.58)	$40.86 (12.28)	$36.06 (7.11)
Albuquerque, NM (0010740)	$31.48 (7.49)	$41.94 (4.86)	$36.06 (8.6)
Allentown-Bethlehem-Easton, PA-NJ (0010900)	$21.81 (6.75)	$39.14 (6.99)	$35.34 (8.14)
Amarillo, TX (0011100)	$19.07 (6.86)	$37.88 (5.67)	$40.54 (7.83)
Anchorage, AK (0011260)	$29.2 (7.56)	$49.59 (13.25)	$49.67 (12.18)
Ann Arbor, MI (0011460)	$24.39 (7.81)	$41.54 (1.88)	$38.78 (6.27)
Arizona nonmetropolitan area (0400001)	$18.66 (8.07)	$39.29 (6.6)	$31.98 (8.14)
Asheville, NC (0011700)	$20.61 (10.55)	$37.98 (5.83)	$37.67 (5.05)
Atlanta-Sandy Springs-Roswell, GA (0012060)	$24.48 (8.61)	$43.4 (8.18)	$40.95 (9.9)
Atlantic City-Hammonton, NJ (0012100)	$19.85 (7.76)	$42.56 (3.05)	$35.34 (7.96)
Austin-Round Rock, TX (0012420)	$20.93 (8.73)	$41.69 (3.48)	$40.54 (12.76)
Bakersfield, CA (0012540)	$21.75 (13.58)	$56.81 (4.89)	$64.1 (6.71)
Balance of Lower Peninsula of Michigan nonmetropolitan area (2600004)	$23.92 (6.13)	$35.95 (6.16)	$38.78 (4.95)
Baltimore-Columbia-Towson, MD (0012580)	$22.4 (8.5)	$43.03 (8.35)	$50.07 (10.01)
Bangor, ME (0070750)	$21.69 (10.01)	$39.95 (3.69)	$50.07 (6.31)
Barnstable Town, MA (0070900)	$25.57 (19.2)	$46.24 (5)	$50.07 (11.35)
Baton Rouge, LA (0012940)	$25.05 (6.61)	$35.71 (14.91)	$36.5 (7.4)
Bellingham, WA (0013380)	$19.6 (8.5)	$45.03 (3.62)	$48.88 (5.37)
Bend-Redmond, OR (0013460)	$31.36 (11.15)	$51.53 (5.8)	$51.26 (10.48)
Billings, MT (0013740)	$21.92 (9.45)	$39.22 (4.71)	$37.67 (10.03)
Binghamton, NY (0013780)	$21.98 (4.76)	$37.81 (5.35)	$36.06 (6.8)
Birmingham-Hoover, AL (0013820)	$19.09 (9.14)	$33.79 (1.39)	$49.67 (5.93)
Bismarck, ND (0013900)	$33.09 (5.53)	$34.99 (14.83)	$37.67 (5.27)
Bloomington, IL (0014010)	$18.52 (6.04)	$35.86 (0.67)	$33.35 (9)
Boise City, ID (0014260)	$27.3 (8.75)	$39.06 (12.72)	$33.35 (7.11)
Border Region of Texas nonmetropolitan area (4800005)	$19.76 (6.9)	$35.06 (3.74)	$40.54 (9.92)
Boston-Cambridge-Nashua, MA-NH (0071650)	$25.39 (18.42)	$51.44 (5.07)	$35.34 (10.43)
Boulder, CO (0014500)	$24.52 (9.24)	$44.57 (6.24)	$41.63 (8.6)
Bremerton-Silverdale, WA (0014740)	$26.7 (9.72)	$46.69 (5.27)	$48.88 (1.02)
Bridgeport-Stamford-Norwalk, CT (0071950)	$25.67 (8.27)	$47.57 (5.6)	$45.32 (7.52)
Brownsville-Harlingen, TX (0015180)	$15.71 (5.87)	$35.07 (3.01)	$40.54 (7.4)
Buffalo-Cheektowaga-Niagara Falls, NY (0015380)	$21.61 (8.34)	$41.55 (5.29)	$36.06 (8)
Burlington-South Burlington, VT (0072400)	$23.29 (7.29)	$39.06 (6.32)	$39.36 (6.86)
Canton-Massillon, OH (0015940)	$21.68 (6.83)	$35.94 (5.06)	$37.72 (5.88)
Cape Coral-Fort Myers, FL (0015980)	$25.87 (2.09)	$38.04 (9.55)	$38.42 (8.43)
Capital/Northern New York nonmetropolitan area (3600001)	$22.09 (6.66)	$38.2 (4.73)	$36.06 (7.51)
Carson City, NV (0016180)	$34.99 (5.96)	$43.63 (20.44)	$36.06 (7.35)
Central East New York nonmetropolitan area (3600006)	$21.39 (6.14)	$37.3 (5.37)	$36.06 (5.35)
Central Indiana nonmetropolitan area (1800002)	$17.33 (6.2)	$34.16 (3.28)	$33.35 (7.49)
Central Kentucky nonmetropolitan area (2100003)	$19.16 (5.32)	$35.15 (4.07)	$37.32 (5.82)
Central Missouri nonmetropolitan area (2900001)	$21.05 (6.43)	$31.69 (3.47)	$34.55 (6.82)
Central Oregon nonmetropolitan area (4100007)	$19.83 (7.44)	$48.35 (3.41)	$51.26 (8.86)
Champaign-Urbana, IL (0016580)	$16.4 (9.31)	$38.13 (1.07)	$33.35 (4.41)
Charleston, WV (0016620)	$19.33 (7.01)	$34.11 (5.51)	$38.94 (8.76)
Charleston-North Charleston, SC (0016700)	$22.09 (5.92)	$36.96 (5.64)	$35.74 (5.86)
Charlotte-Concord-Gastonia, NC-SC (0016740)	$22.23 (7.18)	$38.24 (5.59)	$35.74 (9.2)
Charlottesville, VA (0016820)	$22.01 (8.55)	$38.89 (4.22)	$36.73 (7.46)
Chattanooga, TN-GA (0016860)	$23.21 (5.82)	$34.77 (3.93)	$40.95 (9.74)
Chicago-Naperville-Elgin, IL-IN-WI (0016980)	$22.41 (8.07)	$40.99 (5.2)	$38.94 (12.43)
Chico, CA (0017020)	$22.24 (16.25)	$60.78 (5.45)	$64.1 (9.75)
Cincinnati, OH-KY-IN (0017140)	$22.4 (6.37)	$38.82 (5.2)	$33.35 (10.35)
Cleveland-Elyria, OH (0017460)	$20.06 (3.94)	$38.95 (5.94)	$37.72 (7.1)
Coast Oregon nonmetropolitan area (4100006)	$24.49 (8.25)	$48.16 (6.49)	$51.26 (7.17)
Coastal Plains Region of Texas nonmetropolitan area (4800006)	$17.84 (5.88)	$35.27 (2.38)	$40.54 (6.06)
Coeur d'Alene, ID (0017660)	$20.08 (7.7)	$40.5 (2.82)	$33.35 (9.15)
College Station-Bryan, TX (0017780)	$18.88 (9.85)	$37.59 (2.94)	$40.54 (8.44)
Colorado Springs, CO (0017820)	$22.76 (9.29)	$39.78 (4.72)	$41.63 (10.08)
Columbia, MO (0017860)	$23.01 (9.61)	$34 (9.63)	$34.55 (10.4)
Columbia, SC (0017900)	$22.62 (6.29)	$35.37 (5.72)	$35.74 (6.27)
Columbus, OH (0018140)	$20.78 (6.11)	$38.8 (5.24)	$37.72 (6.37)
Corpus Christi, TX (0018580)	$20.29 (6.49)	$37.04 (5.46)	$40.54 (8.15)
Corvallis, OR (0018700)	$27.6 (9.69)	$53.66 (9.93)	$51.26 (8.03)
Crestview-Fort Walton Beach-Destin, FL (0018880)	$20.29 (6.01)	$35.35 (5.8)	$38.42 (9.15)
Cumberland, MD-WV (0019060)	$17.81 (6.22)	$34.64 (1.85)	$38.94 (4.37)
Dallas-Fort Worth-Arlington, TX (0019100)	$21.03 (9.64)	$42.24 (2.76)	$40.54 (8.2)
Davenport-Moline-Rock Island, IA-IL (0019340)	$23.5 (5.95)	$32.77 (7.97)	$33.35 (10.24)
Dayton, OH (0019380)	$23.83 (6.95)	$38.04 (1.72)	$37.72 (3.45)
Denver-Aurora-Lakewood, CO (0019740)	$25.67 (8.27)	$42.21 (5.46)	$41.63 (6.16)
Des Moines-West Des Moines, IA (0019780)	$22.31 (6.2)	$34.16 (5.17)	$54.43 (6.58)
Detroit-Warren-Dearborn, MI (0019820)	$22.06 (7.68)	$39.42 (4.43)	$38.78 (6.21)
Duluth, MN-WI (0020260)	$17.04 (5.74)	$36.07 (7.78)	$38.94 (5.15)
East Arkansas nonmetropolitan area (0500002)	$19.79 (5.56)	$31.57 (4.57)	$49.67 (7.24)
East Central Illinois nonmetropolitan area (1700003)	$21.73 (5.04)	$34.31 (6.11)	$33.35 (4.32)
East Kentucky nonmetropolitan area (2100004)	$20.6 (5.34)	$33.27 (6.91)	$37.32 (5.64)
East North Dakota nonmetropolitan area (3800007)	$23.44 (5.73)	$34.94 (7.95)	$37.67 (7.45)
East-Central Montana nonmetropolitan area (3000006)	$24.05 (6.43)	$36.42 (9.18)	$37.67 (6.98)
Eastern New Mexico nonmetropolitan area (3500007)	$18.17 (6.6)	$38.23 (3.62)	$36.06 (7.34)
Eastern Oregon nonmetropolitan area (4100008)	$27.27 (8.15)	$46.63 (6.37)	$51.26 (7.98)
Eastern Sierra-Mother Lode Region of California nonmetropolitan area (0600006)	$25.41 (19.21)	$61.45 (7.42)	$64.1 (10.62)
Eastern Washington nonmetropolitan area (5300007)	$21.4 (8.31)	$44.93 (9.09)	$48.88 (7.58)
Eastern and Southern Colorado nonmetropolitan area (0800001)	$19.7 (6.55)	$37.89 (6.47)	$41.63 (2.83)
Eau Claire, WI (0020740)	$22.12 (7.42)	$38.18 (6.54)	$38.94 (11.48)
El Centro, CA (0020940)	$29.69 (8.15)	$47.73 (23.14)	$64.1 (7.8)
El Paso, TX (0021340)	$16.42 (6.69)	$36.36 (0)	$40.54 (7.88)
Elkhart-Goshen, IN (0021140)	$20.1 (6.29)	$34.58 (4.66)	$33.35 (5.62)
Erie, PA (0021500)	$20.03 (5.56)	$35 (7.3)	$38.76 (6.8)
Eugene, OR (0021660)	$24.12 (6.82)	$47.54 (8.67)	$51.26 (6.26)
Evansville, IN-KY (0021780)	$20.91 (7.85)	$35.16 (6.38)	$37.32 (4.74)
Fairbanks, AK (0021820)	$32.29 (7.56)	$49.14 (13.25)	$49.67 (5.01)
Fargo, ND-MN (0022020)	$25.11 (6.21)	$36.6 (10.27)	$42.72 (6.15)
Fayetteville, NC (0022180)	$19.02 (10.18)	$38.3 (5.06)	$37.67 (8.05)
Flint, MI (0022420)	$24.75 (8.09)	$41.61 (6.87)	$38.78 (9.76)
Florence, SC (0022500)	$21.33 (7.61)	$33.68 (3.54)	$35.74 (5.42)
Fort Collins, CO (0022660)	$26.73 (7.12)	$40.85 (7.23)	$41.63 (15.59)
Fort Wayne, IN (0023060)	$21.54 (6.16)	$35.21 (5.44)	$33.35 (0.58)
Fresno, CA (0023420)	$24.54 (16.69)	$59.31 (10.34)	$64.1 (4.48)
Gainesville, FL (0023540)	$20.93 (11.74)	$38.91 (6.62)	$38.42 (7.73)
Grand Forks, ND-MN (0024220)	$24.78 (5.97)	$34.81 (13.85)	$42.72 (5.72)
Grand Junction, CO (0024300)	$18.17 (10.51)	$40.36 (3.6)	$41.63 (4.58)
Grand Rapids-Wyoming, MI (0024340)	$22.39 (5.38)	$36.98 (4.62)	$38.78 (5.75)
Green Bay, WI (0024580)	$25.03 (5.09)	$37.41 (6.33)	$38.94 (8.02)
Greensboro-High Point, NC (0024660)	$24.32 (11.31)	$38.59 (2.96)	$37.67 (8.09)
Greenville-Anderson-Mauldin, SC (0024860)	$21.47 (6.58)	$35.83 (3.21)	$35.74 (8.21)
Gulfport-Biloxi-Pascagoula, MS (0025060)	$18.35 (5.91)	$34.3 (4.23)	$34.55 (7.98)
Hagerstown-Martinsburg, MD-WV (0025180)	$20.48 (8.69)	$38.73 (3.99)	$38.94 (10.94)
Harrisburg-Carlisle, PA (0025420)	$21.93 (7.23)	$38.39 (4.68)	$38.76 (9.43)
Hartford-West Hartford-East Hartford, CT (0073450)	$28.31 (7.9)	$43.72 (8.09)	$45.32 (3.99)
Hawaii / Kauai nonmetropolitan area (1500001)	$23.04 (13.14)	$50.57 (12.36)	$54.43 (7.18)
Hickory-Lenoir-Morganton, NC (0025860)	$20.82 (5.92)	$33.84 (5.69)	$37.67 (7.43)
Hill Country Region of Texas nonmetropolitan area (4800004)	$19.06 (6.4)	$37.17 (3.01)	$40.54 (9.68)
Hilton Head Island-Bluffton-Beaufort, SC (0025940)	$25.57 (5.91)	$32.62 (6.8)	$35.74 (7.37)
Houston-The Woodlands-Sugar Land, TX (0026420)	$21.81 (8.67)	$42.73 (4.32)	$40.54 (6.77)
Huntington-Ashland, WV-KY-OH (0026580)	$14.81 (7.58)	$35.8 (6.52)	$37.72 (5.89)
Indianapolis-Carmel-Anderson, IN (0026900)	$21.64 (7.44)	$38.68 (3.65)	$33.35 (6.68)
Jackson, MS (0027140)	$19.15 (6.98)	$33.53 (6.92)	$34.55 (8.92)
Jacksonville, FL (0027260)	$22.88 (6.37)	$37.74 (8.71)	$38.42 (10.75)
Kahului-Wailuku-Lahaina, HI (0027980)	$17.87 (14.91)	$52.81 (2.64)	$54.43 (9.5)
Kalamazoo-Portage, MI (0028020)	$24.61 (6.34)	$37.02 (5.03)	$38.78 (9.51)
Kansas City, MO-KS (0028140)	$22.26 (7.13)	$36.82 (7.39)	$34.61 (5.01)
Kennewick-Richland, WA (0028420)	$23.06 (7.23)	$42.96 (5.78)	$48.88 (8.54)
Killeen-Temple, TX (0028660)	$20.84 (8.91)	$39.44 (3.06)	$40.54 (9.7)
Knoxville, TN (0028940)	$19.12 (7.98)	$31.99 (3.17)	$34.85 (5.92)
Lafayette-West Lafayette, IN (0029200)	$19.21 (6.55)	$35.34 (7.18)	$33.35 (10.8)
Lancaster, PA (0029540)	$22.06 (6.53)	$37.16 (3.67)	$38.76 (5.04)
Lansing-East Lansing, MI (0029620)	$26.57 (8.04)	$39.07 (9.36)	$38.78 (8.46)
Laredo, TX (0029700)	$17.09 (6.07)	$36.73 (3.56)	$40.54 (10.3)
Las Cruces, NM (0029740)	$17.5 (6.39)	$37.3 (3.45)	$36.06 (6.62)
Las Vegas-Henderson-Paradise, NV (0029820)	$25.36 (8.94)	$46.96 (1.93)	$36.06 (6.12)
Lexington-Fayette, KY (0030460)	$20.39 (6.12)	$37.36 (7.62)	$37.32 (7.59)
Lima, OH (0030620)	$21.53 (5.46)	$35.12 (4.22)	$37.72 (5.65)
Lincoln, NE (0030700)	$23.25 (6.79)	$35.52 (6.49)	$35.34 (5.02)
Little Rock-North Little Rock-Conway, AR (0030780)	$21.57 (6.07)	$34.48 (2.52)	$49.67 (13.82)
Longview, WA (0031020)	$22.98 (11.9)	$46.28 (7.44)	$48.88 (8.46)
Los Angeles-Long Beach-Anaheim, CA (0031080)	$24.66 (14.1)	$60.26 (6.69)	$64.1 (5.04)
Louisville/Jefferson County, KY-IN (0031140)	$27.85 (6.81)	$39.08 (0.23)	$33.35 (8.87)
Lubbock, TX (0031180)	$18.83 (6.54)	$37.36 (3.02)	$40.54 (4.57)
Madison, WI (0031540)	$19.44 (6.91)	$41.46 (6.71)	$38.94 (5.26)
Manchester, NH (0074950)	$27.83 (10.96)	$39.38 (12.28)	$35.34 (9.13)
Mansfield, OH (0031900)	$16.36 (5.17)	$36.26 (2.15)	$37.72 (16.2)
Maryland nonmetropolitan area (2400006)	$20.87 (7.51)	$36.72 (5.74)	$50.07 (5.66)
Massachusetts nonmetropolitan area (2500006)	$23.91 (9.19)	$45.1 (8.27)	$50.07 (7.69)
McAllen-Edinburg-Mission, TX (0032580)	$17.94 (6.1)	$36.44 (4.23)	$40.54 (8.35)
Medford, OR (0032780)	$26.15 (6)	$47.71 (7.92)	$51.26 (14.61)
Memphis, TN-MS-AR (0032820)	$23.03 (6.38)	$35.8 (8.89)	$49.67 (8.64)
Merced, CA (0032900)	$20.09 (13.83)	$56.6 (3.73)	$64.1 (6.21)
Miami-Fort Lauderdale-West Palm Beach, FL (0033100)	$24.16 (8.45)	$39.33 (9.47)	$38.42 (7.96)
Milwaukee-Waukesha-West Allis, WI (0033340)	$23.02 (8.09)	$39.44 (5.95)	$38.94 (5.31)
Minneapolis-St. Paul-Bloomington, MN-WI (0033460)	$23.81 (8.62)	$44.32 (4.06)	$38.94 (6.14)
Missoula, MT (0033540)	$22.38 (7.33)	$37.93 (6.77)	$37.67 (14)
Mobile, AL (0033660)	$19.17 (7.73)	$31.41 (1.65)	$49.67 (6.65)
Modesto, CA (0033700)	$26.64 (20.85)	$66.59 (9.81)	$64.1 (7.61)
Montgomery, AL (0033860)	$18.18 (6.56)	$33.98 (2.95)	$49.67 (5.43)
Mount Vernon-Anacortes, WA (0034580)	$23.69 (9.24)	$45.87 (6.74)	$48.88 (22.06)
Mountain North Carolina nonmetropolitan area (3700004)	$18.47 (5.29)	$34.06 (3.84)	$37.67 (10.4)
Napa, CA (0034900)	$37.99 (14.37)	$70.38 (14.71)	$64.1 (6.88)
Naples-Immokalee-Marco Island, FL (0034940)	$20.35 (10.1)	$39.56 (3.95)	$38.42 (9.35)
Nashville-Davidson–Murfreesboro–Franklin, TN (0034980)	$24.56 (7.06)	$37.13 (4.49)	$34.85 (6.63)
Nevada nonmetropolitan area (3200006)	$22.8 (7.82)	$42.65 (2.56)	$36.06 (4.43)
New Bedford, MA (0075550)	$22.96 (9.99)	$43.67 (3.6)	$50.07 (11.08)
New Haven, CT (0075700)	$29.27 (7.01)	$46.18 (9.18)	$45.32 (12)
New Orleans-Metairie, LA (0035380)	$19.67 (6.64)	$38.18 (3.63)	$36.5 (6.79)
New York-Newark-Jersey City, NY-NJ-PA (0035620)	$27.1 (7.84)	$50.41 (6.89)	$38.76 (6.09)
North Coast Region of California nonmetropolitan area (0600003)	$21.92 (12.78)	$58.05 (3.71)	$64.1 (8.97)
North Missouri nonmetropolitan area (2900002)	$19.2 (6.89)	$31.79 (2.54)	$34.55 (6.65)
North Northeastern Ohio nonmetropolitan area (3900002)	$19.09 (4.98)	$33.95 (4.37)	$37.72 (14.55)
North Port-Sarasota-Bradenton, FL (0035840)	$22.06 (6.26)	$38.03 (5.33)	$38.42 (5.75)
North Texas Region of Texas nonmetropolitan area (4800002)	$21.14 (7.32)	$37.4 (4.08)	$40.54 (5.9)
North Valley-Northern Mountains Region of California nonmetropolitan area (0600007)	$23.38 (12.66)	$59.35 (7.15)	$64.1 (5.88)
Northeast Coastal North Carolina nonmetropolitan area (3700002)	$20.3 (6.48)	$34.48 (2.62)	$37.67 (7.38)
Northeast Lower Peninsula of Michigan nonmetropolitan area (2600002)	$21.66 (5.5)	$35.3 (2.47)	$38.78 (8.44)
Northeast Mississippi nonmetropolitan area (2800001)	$16.22 (5.75)	$31.58 (5.09)	$34.55 (5.88)
Northeast Oklahoma nonmetropolitan area (4000001)	$17.77 (6.61)	$35.96 (5.34)	$36.98 (7.1)
Northeast South Carolina nonmetropolitan area (4500006)	$23.19 (6.35)	$35.27 (11.04)	$35.74 (7.54)
Northeastern Wisconsin nonmetropolitan area (5500002)	$23.92 (6.32)	$36.8 (4.08)	$38.94 (5.42)
Northern Indiana nonmetropolitan area (1800001)	$19.92 (5.36)	$32.66 (6.63)	$33.35 (5.66)
Northern New Mexico nonmetropolitan area (3500006)	$19.17 (9.26)	$40.28 (2.41)	$36.06 (7.42)
Northern Pennsylvania nonmetropolitan area (4200002)	$19.87 (5.17)	$35.13 (2.55)	$38.76 (11.92)
Northern Vermont nonmetropolitan area (5000002)	$20.19 (6.03)	$37.28 (6.81)	$39.36 (7.75)
Northern West Virginia nonmetropolitan area (5400002)	$17.87 (6.22)	$33.21 (2.39)	$38.94 (5.51)
Northwest Colorado nonmetropolitan area (0800003)	$22.17 (7.58)	$43.06 (4.22)	$41.63 (4.37)
Northwest Illinois nonmetropolitan area (1700001)	$19.43 (6.03)	$35.97 (4.94)	$33.35 (8.82)
Northwest Lower Peninsula of Michigan nonmetropolitan area (2600003)	$21.16 (5.77)	$35.6 (2.98)	$38.78 (6.19)
Northwest Minnesota nonmetropolitan area (2700001)	$22.07 (8.98)	$39.22 (5.8)	$42.72 (6.93)
Northwest Mississippi nonmetropolitan area (2800002)	$18.46 (6.22)	$32.01 (6.66)	$34.55 (7.35)
Northwestern Idaho nonmetropolitan area (1600006)	$20.99 (6.32)	$37.31 (4.32)	$33.35 (8.32)
Ogden-Clearfield, UT (0036260)	$20.2 (6.45)	$35.16 (1.5)	$36.73 (6.15)
Oklahoma City, OK (0036420)	$22.52 (6.58)	$37.19 (7.23)	$36.98 (7.32)
Olympia-Tumwater, WA (0036500)	$22.67 (9.22)	$46.89 (5.74)	$48.88 (10.9)
Omaha-Council Bluffs, NE-IA (0036540)	$21.13 (5.45)	$36.18 (8.1)	$54.43 (6.04)
Orlando-Kissimmee-Sanford, FL (0036740)	$21.96 (6.44)	$38.04 (8.96)	$38.42 (7.48)
Oxnard-Thousand Oaks-Ventura, CA (0037100)	$24.62 (12.92)	$58.78 (6.16)	$64.1 (9.65)
Palm Bay-Melbourne-Titusville, FL (0037340)	$23.8 (6.17)	$35.61 (12.89)	$38.42 (7.8)
Pensacola-Ferry Pass-Brent, FL (0037860)	$21.32 (6.04)	$34.38 (9.11)	$38.42 (9.19)
Peoria, IL (0037900)	$19.95 (4.29)	$34.93 (5.66)	$33.35 (5.15)
Philadelphia-Camden-Wilmington, PA-NJ-DE-MD (0037980)	$23.06 (7.62)	$42.23 (5.17)	$50.07 (8.29)
Phoenix-Mesa-Scottsdale, AZ (0038060)	$22.75 (7.83)	$42.03 (5.11)	$31.98 (10.29)
Piedmont North Carolina nonmetropolitan area (3700003)	$20.02 (6.12)	$35.32 (6.25)	$37.67 (4.55)
Pittsburgh, PA (0038300)	$21.54 (6.72)	$36.65 (2.59)	$38.76 (9.63)
Port St. Lucie, FL (0038940)	$21.84 (6.3)	$37.13 (5.16)	$38.42 (9.01)
Portland-South Portland, ME (0076750)	$25.26 (6.53)	$38.13 (6.77)	$50.07 (13.76)
Portland-Vancouver-Hillsboro, OR-WA (0038900)	$27.93 (9.69)	$53.66 (10.48)	$48.88 (7.2)
Providence-Warwick, RI-MA (0077200)	$28.53 (9.25)	$42.39 (13.21)	$50.07 (6.29)
Provo-Orem, UT (0039340)	$21.57 (6.05)	$34.96 (2.22)	$36.73 (9.01)
Raleigh, NC (0039580)	$25.32 (6.66)	$37.82 (0.01)	$37.67 (8.13)
Reading, PA (0039740)	$18.61 (9.99)	$38.64 (4.74)	$38.76 (11.74)
Reno, NV (0039900)	$29.24 (7.65)	$44.85 (10.01)	$36.06 (6.18)
Richmond, VA (0040060)	$27.13 (9.24)	$39.75 (3.85)	$36.73 (7.74)
Riverside-San Bernardino-Ontario, CA (0040140)	$26.25 (14.31)	$58.4 (9.14)	$64.1 (10.26)
Roanoke, VA (0040220)	$21.81 (5.49)	$35.94 (3.7)	$36.73 (5.82)
Rochester, MN (0040340)	$26.97 (6.12)	$43.3 (7.35)	$42.72 (17.87)
Rochester, NY (0040380)	$22.11 (9.99)	$40.33 (4.76)	$36.06 (12.82)
Rockford, IL (0040420)	$20.33 (6.16)	$37.11 (4.34)	$33.35 (11.22)
Sacramento–Roseville–Arden-Arcade, CA (0040900)	$30.51 (22.12)	$69.82 (9.63)	$64.1 (7.46)
Salem, OR (0041420)	$23.7 (8.03)	$46.17 (7.82)	$51.26 (6.05)
Salinas, CA (0041500)	$23.83 (11.13)	$58.19 (5.77)	$64.1 (9.6)
Salisbury, MD-DE (0041540)	$21.64 (9.76)	$39.54 (6.36)	$47.23 (14.47)
Salt Lake City, UT (0041620)	$24.43 (6.84)	$38.16 (7.14)	$36.73 (16.68)
San Antonio-New Braunfels, TX (0041700)	$20.3 (10.55)	$39.92 (2.99)	$40.54 (18.21)
San Diego-Carlsbad, CA (0041740)	$24.97 (14.9)	$56.65 (6.94)	$64.1 (4.85)
San Francisco-Oakland-Hayward, CA (0041860)	$32.51 (17.89)	$79.21 (10.27)	$64.1 (13.95)
San Jose-Sunnyvale-Santa Clara, CA (0041940)	$33.04 (17.8)	$76.94 (7.02)	$64.1 (13.37)
San Juan-Carolina-Caguas, PR (0041980)	$16.77 (3.36)	$18.69 (5.38)	$17.96 (12.01)
San Luis Obispo-Paso Robles-Arroyo Grande, CA (0042020)	$31.12 (11.93)	$60.31 (12.58)	$64.1 (20.54)
Santa Cruz-Watsonville, CA (0042100)	$25.44 (20.44)	$72 (3.43)	$64.1 (5.85)
Santa Maria-Santa Barbara, CA (0042200)	$26.53 (14.28)	$55.37 (9.79)	$64.1 (11.72)
Santa Rosa, CA (0042220)	$28.99 (19.2)	$72.67 (10.25)	$64.1 (4.27)
Scranton–Wilkes-Barre–Hazleton, PA (0042540)	$19.47 (6)	$35.35 (4.11)	$38.76 (8.01)
Seattle-Tacoma-Bellevue, WA (0042660)	$25.41 (14.74)	$50.74 (7.92)	$48.88 (4.98)
Sioux City, IA-NE-SD (0043580)	$22.89 (5.84)	$32.1 (5.82)	$31.01 (9.26)
South Central Kentucky nonmetropolitan area (2100002)	$18.49 (6.12)	$34.29 (3.85)	$37.32 (5.52)
South Central Wisconsin nonmetropolitan area (5500003)	$22.42 (5.67)	$37.11 (9.63)	$38.94 (4.07)
South Illinois nonmetropolitan area (1700004)	$18.19 (5.74)	$34.03 (3.9)	$33.35 (7.96)
South Nebraska nonmetropolitan area (3100006)	$19.22 (5.54)	$33.72 (5.24)	$35.34 (6.14)
Southeast Coastal North Carolina nonmetropolitan area (3700001)	$19.87 (6)	$34.07 (4.66)	$37.67 (7.33)
Southeast Minnesota nonmetropolitan area (2700004)	$18.91 (8.81)	$40.11 (6.23)	$42.72 (8.49)
Southeast Mississippi nonmetropolitan area (2800003)	$15.41 (4.77)	$29.75 (3.57)	$34.55 (5.93)
Southeast Missouri nonmetropolitan area (2900003)	$19.51 (6.68)	$31.58 (7.8)	$34.55 (5.35)
Southeast Oklahoma nonmetropolitan area (4000004)	$18.46 (6.38)	$34.11 (5.26)	$36.98 (3.8)
Southern Indiana nonmetropolitan area (1800003)	$18.87 (5.46)	$32.22 (4.24)	$33.35 (8.29)
Southern Ohio nonmetropolitan area (3900004)	$21.54 (5.69)	$35.42 (5.69)	$37.72 (4.52)
Southern West Virginia nonmetropolitan area (5400001)	$13.96 (7.8)	$31.36 (5.57)	$38.94 (4.15)
Southwest Colorado nonmetropolitan area (0800002)	$23.58 (5.69)	$39.2 (3.87)	$41.63 (7.7)
Southwest Iowa nonmetropolitan area (1900003)	$20.25 (4.25)	$31.67 (6.55)	$54.43 (10.77)
Southwest Maine nonmetropolitan area (2300002)	$20.87 (5.72)	$36.43 (2.19)	$50.07 (5.61)
Southwest Mississippi nonmetropolitan area (2800004)	$15.62 (6.53)	$31.41 (3.79)	$34.55 (6.32)
Southwest Missouri nonmetropolitan area (2900004)	$19.35 (3.56)	$30.37 (5.2)	$34.55 (9.6)
Southwest Montana nonmetropolitan area (3000003)	$22.41 (7.4)	$38.2 (6.12)	$37.67 (10.51)
Southwest New York nonmetropolitan area (3600004)	$23.72 (6.15)	$36.11 (8.08)	$36.06 (8.03)
Spokane-Spokane Valley, WA (0044060)	$20.96 (9.84)	$47.16 (5.26)	$48.88 (7.18)
Springfield, IL (0044100)	$24.79 (9.65)	$37.8 (7.29)	$33.35 (5.68)
Springfield, MA-CT (0078100)	$20.67 (10.48)	$42.04 (3.65)	$45.32 (7.73)
Springfield, MO (0044180)	$20.94 (8.35)	$30.53 (5.66)	$34.55 (10.46)
St. Cloud, MN (0041060)	$20.86 (8.54)	$43.96 (3.97)	$42.72 (7.42)
St. Louis, MO-IL (0041180)	$22.36 (7.23)	$36.14 (6.64)	$33.35 (3.07)
Stockton-Lodi, CA (0044700)	$23.93 (12.56)	$62.68 (7.03)	$64.1 (5.53)
Syracuse, NY (0045060)	$23.64 (9.29)	$37.66 (5.83)	$36.06 (7.62)
Tallahassee, FL (0045220)	$20.07 (6)	$34.56 (3.67)	$38.42 (4.76)
Tampa-St. Petersburg-Clearwater, FL (0045300)	$23.25 (6.55)	$38.42 (8.9)	$38.42 (6.02)
Terre Haute, IN (0045460)	$17.78 (5.83)	$32.81 (5.89)	$33.35 (8.33)
Toledo, OH (0045780)	$22.13 (5.7)	$36.8 (8.56)	$37.72 (6.57)
Trenton, NJ (0045940)	$21.09 (9.54)	$44.02 (3.1)	$35.34 (6.19)
Tucson, AZ (0046060)	$20.15 (6.45)	$40.19 (6)	$31.98 (4.79)
Tulsa, OK (0046140)	$20.87 (6.38)	$38.32 (6.47)	$36.98 (9.18)
Tyler, TX (0046340)	$19.42 (6.5)	$35.96 (1.81)	$40.54 (7.63)
Upper Peninsula of Michigan nonmetropolitan area (2600001)	$19.41 (5.8)	$34.94 (4.93)	$38.78 (19.82)
Urban Honolulu, HI (0046520)	$17.57 (13.34)	$55.38 (3.21)	$54.43 (9.32)
Utica-Rome, NY (0046540)	$21.7 (5.44)	$38.11 (4.8)	$36.06 (11.43)
Vallejo-Fairfield, CA (0046700)	$30.01 (19.64)	$76.12 (9.55)	$64.1 (5.58)
Virginia Beach-Norfolk-Newport News, VA-NC (0047260)	$23.56 (7.29)	$38.2 (6.82)	$37.67 (7.74)
Visalia-Porterville, CA (0047300)	$25.68 (12.35)	$54.77 (9.35)	$64.1 (4.43)
Waco, TX (0047380)	$22.47 (9.39)	$38.36 (5.39)	$40.54 (5.78)
Washington-Arlington-Alexandria, DC-VA-MD-WV (0047900)	$25.87 (8.99)	$44.61 (6.46)	$38.94 (7.62)
Waterloo-Cedar Falls, IA (0047940)	$23.17 (5.19)	$32.57 (2.7)	$54.43 (7.5)
Watertown-Fort Drum, NY (0048060)	$19.05 (9.6)	$40.26 (4.93)	$36.06 (5.89)
West Central Illinois nonmetropolitan area (1700002)	$22.21 (4.97)	$34.16 (4.94)	$33.35 (7.63)
West Central-Southwest New Hampshire nonmetropolitan area (3300006)	$20.79 (9.48)	$40.1 (5.01)	$35.34 (9.41)
West Kentucky nonmetropolitan area (2100001)	$22.76 (5.58)	$34.11 (3.25)	$37.32 (9.79)
West Montana nonmetropolitan area (3000004)	$22.63 (6.94)	$36.23 (4.05)	$37.67 (3.91)
West Texas Region of Texas nonmetropolitan area (4800001)	$17.58 (6.28)	$36.81 (2.91)	$40.54 (10.8)
Western Washington nonmetropolitan area (5300006)	$22.51 (9.04)	$46.71 (5.72)	$48.88 (8.35)
Western Wisconsin nonmetropolitan area (5500004)	$24.04 (6.52)	$39.04 (8.71)	$38.94 (6.31)
Western Wyoming nonmetropolitan area (5600006)	$23.19 (6.32)	$36.94 (8)	$38.95 (8.57)
Wichita Falls, TX (0048660)	$18.31 (6.17)	$35.64 (1.83)	$40.54 (8.42)
Wichita, KS (0048620)	$21.89 (5.74)	$33.08 (4.73)	$34.61 (7.28)
Wilmington, NC (0048900)	$21.03 (6.92)	$36.27 (5.79)	$37.67 (4.63)
Winston-Salem, NC (0049180)	$22.84 (10.89)	$38.57 (2.65)	$37.67 (5.96)
Worcester, MA-CT (0079600)	$22.35 (17.27)	$47.38 (5.29)	$45.32 (4.56)
Yakima, WA (0049420)	$23.56 (9.55)	$42.98 (6.48)	$48.88 (14.99)
York-Hanover, PA (0049620)	$19.87 (7.1)	$36.8 (5.74)	$38.76 (5.74)
Youngstown-Warren-Boardman, OH-PA (0049660)	$21.01 (5.31)	$34.58 (5.69)	$38.76 (5.69)
Yuma, AZ (0049740)	$17.56 (8.91)	$41.19 (5.79)	$31.98 (5.79)

Additional input data across the domains of equipment, software, transportation, space, and benefits can be downloaded in spreadsheet format from: https://github.com/sanjaybasu/chw_payment

**Table 5:** Estimated minimum payment threshold rates for sustaining community health worker (CHW) programs across metropolitan and non-metropolitan areas

Area (census statistical area code number)	Minimum payment per visit	95% CI (FFS lower)	95% CI (FFS upper)	Minimum payment per month per patient	95% CI (PMPM lower)	95% CI (PMPM upper)
Abilene, TX (0010180)	$49.36	$24.51	$82.74	$132.24	$96.63	$259.02
Akron, OH (0010420)	$53.41	$25.64	$90.24	$139.43	$104.66	$259.71
Alaska nonmetropolitan area (0200006)	$59.30	$23.36	$105.31	$156.80	$125.57	$271.88
Albany-Schenectady-Troy, NY (0010580)	$50.59	$20.20	$90.97	$138.01	$109.43	$241.70
Albuquerque, NM (0010740)	$52.45	$20.06	$95.81	$142.54	$113.35	$254.05
Allentown-Bethlehem-Easton, PA-NJ (0010900)	$48.69	$22.86	$82.88	$130.12	$97.20	$248.95
Amarillo, TX (0011100)	$49.61	$24.81	$82.48	$131.82	$96.72	$252.70
Anchorage, AK (0011260)	$56.36	$22.85	$100.61	$150.23	$118.97	$263.69
Ann Arbor, MI (0011460)	$55.10	$25.18	$94.86	$144.95	$110.33	$265.42
Arizona nonmetropolitan area (0400001)	$43.43	$21.37	$72.29	$117.44	$85.71	$233.19
Asheville, NC (0011700)	$44.24	$19.20	$77.42	$119.95	$91.43	$225.63
Atlanta-Sandy Springs-Roswell, GA (0012060)	$57.15	$28.18	$97.16	$149.26	$111.54	$274.40
Atlantic City-Hammonton, NJ (0012100)	$47.29	$22.73	$80.86	$126.91	$93.92	$242.25
Austin-Round Rock, TX (0012420)	$52.04	$25.19	$88.26	$136.78	$101.48	$260.15
Bakersfield, CA (0012540)	$62.07	$30.26	$105.59	$157.98	$119.88	$280.43
Balance of Lower Peninsula of Michigan nonmetropolitan area (2600004)	$54.12	$24.92	$92.97	$142.22	$108.17	$261.77
Baltimore-Columbia-Towson, MD (0012580)	$60.37	$28.45	$102.69	$155.02	$118.06	$281.51
Bangor, ME (0070750)	$59.21	$28.00	$100.16	$152.88	$115.92	$280.58
Barnstable Town, MA (0070900)	$63.28	$28.92	$109.42	$163.29	$125.67	$292.25
Baton Rouge, LA (0012940)	$53.86	$25.89	$91.65	$140.41	$105.28	$256.30
Bellingham, WA (0013380)	$53.64	$24.97	$92.58	$141.58	$106.15	$266.73
Bend-Redmond, OR (0013460)	$60.53	$25.90	$105.96	$158.92	$125.00	$278.61
Billings, MT (0013740)	$45.30	$19.19	$80.39	$123.66	$94.63	$233.27
Binghamton, NY (0013780)	$43.84	$18.65	$78.52	$121.44	$92.57	$226.83
Birmingham-Hoover, AL (0013820)	$45.90	$20.41	$79.48	$125.18	$93.98	$243.60
Bismarck, ND (0013900)	$54.29	$20.76	$99.07	$146.28	$116.58	$263.21
Bloomington, IL (0014010)	$42.46	$20.27	$71.19	$116.37	$85.29	$235.05
Boise City, ID (0014260)	$50.25	$21.72	$87.65	$136.40	$104.12	$256.24
Border Region of Texas nonmetropolitan area (4800005)	$50.23	$24.73	$83.90	$132.76	$97.30	$256.21
Boston-Cambridge-Nashua, MA-NH (0071650)	$53.05	$23.98	$92.45	$140.70	$107.61	$257.32
Boulder, CO (0014500)	$54.91	$24.47	$95.48	$145.06	$112.04	$258.58
Bremerton-Silverdale, WA (0014740)	$60.15	$26.88	$104.08	$156.06	$121.47	$274.18
Bridgeport-Stamford-Norwalk, CT (0071950)	$61.26	$27.76	$105.19	$157.14	$121.79	$280.68
Brownsville-Harlingen, TX (0015180)	$46.38	$23.94	$76.11	$124.39	$89.25	$247.10
Buffalo-Cheektowaga-Niagara Falls, NY (0015380)	$44.05	$18.55	$78.33	$121.51	$92.84	$229.51
Burlington-South Burlington, VT (0072400)	$57.67	$27.89	$97.18	$149.68	$112.63	$275.52
Canton-Massillon, OH (0015940)	$52.00	$25.18	$88.34	$137.20	$101.92	$261.82
Cape Coral-Fort Myers, FL (0015980)	$57.24	$27.35	$97.32	$148.09	$111.86	$271.16
Capital/Northern New York nonmetropolitan area (3600001)	$43.82	$18.33	$77.80	$122.19	$93.25	$235.13
Carson City, NV (0016180)	$55.95	$20.68	$102.31	$149.86	$120.92	$261.14
Central East New York nonmetropolitan area (3600006)	$43.36	$18.52	$76.61	$120.18	$91.30	$225.60
Central Indiana nonmetropolitan area (1800002)	$41.34	$20.19	$69.63	$113.07	$82.33	$225.45
Central Kentucky nonmetropolitan area (2100003)	$47.13	$24.25	$77.45	$124.97	$90.53	$240.57
Central Missouri nonmetropolitan area (2900001)	$48.54	$23.76	$81.97	$128.24	$94.33	$242.98
Central Oregon nonmetropolitan area (4100007)	$50.24	$23.80	$85.84	$133.65	$100.03	$248.37
Champaign-Urbana, IL (0016580)	$40.91	$19.84	$69.56	$112.75	$81.26	$230.41
Charleston, WV (0016620)	$47.65	$22.55	$80.41	$127.92	$95.03	$249.76
Charleston-North Charleston, SC (0016700)	$51.14	$25.01	$86.19	$135.09	$100.08	$257.49
Charlotte-Concord-Gastonia, NC-SC (0016740)	$51.51	$24.91	$87.38	$135.80	$100.18	$261.87
Charlottesville, VA (0016820)	$47.65	$20.93	$82.67	$128.36	$97.52	$243.68
Chattanooga, TN-GA (0016860)	$55.33	$27.67	$91.99	$143.56	$106.61	$268.62
Chicago-Naperville-Elgin, IL-IN-WI (0016980)	$51.03	$23.25	$88.30	$136.29	$103.09	$257.35
Chico, CA (0017020)	$63.01	$30.56	$106.00	$159.92	$122.01	$281.16
Cincinnati, OH-KY-IN (0017140)	$46.16	$20.98	$79.52	$125.40	$93.91	$243.96
Cleveland-Elyria, OH (0017460)	$51.07	$25.17	$85.75	$134.06	$99.29	$252.69
Coast Oregon nonmetropolitan area (4100006)	$54.30	$24.65	$94.31	$143.30	$109.25	$259.90
Coastal Plains Region of Texas nonmetropolitan area (4800006)	$48.51	$24.54	$80.53	$128.41	$93.52	$249.51
Coeur d'Alene, ID (0017660)	$44.22	$20.37	$75.31	$121.75	$89.81	$245.23
College Station-Bryan, TX (0017780)	$49.70	$24.75	$83.22	$131.20	$96.31	$251.50
Colorado Springs, CO (0017820)	$52.77	$23.51	$92.26	$140.91	$107.17	$262.68
Columbia, MO (0017860)	$50.36	$24.11	$85.20	$133.01	$99.14	$253.60
Columbia, SC (0017900)	$51.53	$25.06	$86.38	$135.67	$100.67	$259.33
Columbus, OH (0018140)	$51.47	$25.29	$86.84	$135.86	$100.87	$254.65
Corpus Christi, TX (0018580)	$50.65	$24.94	$84.92	$134.59	$99.27	$257.17
Corvallis, OR (0018700)	$57.06	$25.36	$99.55	$151.93	$117.77	$268.42
Crestview-Fort Walton Beach-Destin, FL (0018880)	$51.74	$26.06	$86.04	$135.96	$99.91	$257.59
Cumberland, MD-WV (0019060)	$46.45	$22.54	$78.14	$124.63	$91.85	$239.89
Dallas-Fort Worth-Arlington, TX (0019100)	$52.61	$25.23	$88.24	$136.51	$101.95	$261.01
Davenport-Moline-Rock Island, IA-IL (0019340)	$46.61	$20.79	$79.54	$126.54	$94.72	$249.19
Dayton, OH (0019380)	$54.59	$25.72	$93.31	$141.86	$106.71	$267.03
Denver-Aurora-Lakewood, CO (0019740)	$55.60	$24.21	$97.97	$147.16	$113.44	$269.28
Des Moines-West Des Moines, IA (0019780)	$54.26	$23.27	$95.27	$145.51	$111.77	$269.24
Detroit-Warren-Dearborn, MI (0019820)	$52.73	$24.95	$90.10	$139.08	$105.41	$253.55
Duluth, MN-WI (0020260)	$46.02	$22.09	$76.96	$123.43	$90.59	$244.40
East Arkansas nonmetropolitan area (0500002)	$46.74	$20.70	$80.48	$125.49	$94.76	$239.57
East Central Illinois nonmetropolitan area (1700003)	$45.03	$20.84	$77.96	$122.91	$91.13	$238.73
East Kentucky nonmetropolitan area (2100004)	$48.13	$24.15	$79.57	$128.29	$92.98	$255.68
East North Dakota nonmetropolitan area (3800007)	$45.91	$18.93	$81.58	$126.49	$96.61	$246.78
East-Central Montana nonmetropolitan area (3000006)	$46.96	$19.08	$83.91	$127.42	$98.07	$246.43
Eastern New Mexico nonmetropolitan area (3500007)	$40.74	$17.79	$71.31	$113.55	$84.98	$224.11
Eastern Oregon nonmetropolitan area (4100008)	$56.57	$24.96	$98.84	$148.55	$115.05	$268.92
Eastern Sierra-Mother Lode Region of California nonmetropolitan area (0600006)	$65.83	$31.37	$111.54	$166.94	$128.78	$288.80
Eastern Washington nonmetropolitan area (5300007)	$54.89	$25.45	$94.01	$145.62	$110.09	$269.08
Eastern and Southern Colorado nonmetropolitan area (0800001)	$49.83	$23.12	$85.77	$133.56	$100.30	$252.44
Eau Claire, WI (0020740)	$50.56	$23.35	$86.17	$134.77	$101.66	$253.78
El Centro, CA (0020940)	$67.90	$31.79	$115.90	$172.56	$134.26	$296.22
El Paso, TX (0021340)	$47.43	$23.96	$78.63	$126.20	$90.62	$253.02
Elkhart-Goshen, IN (0021140)	$43.86	$20.21	$75.07	$119.71	$88.04	$244.00
Erie, PA (0021500)	$53.66	$26.42	$89.53	$139.83	$104.01	$265.39
Eugene, OR (0021660)	$53.31	$24.25	$91.57	$143.21	$109.24	$266.07
Evansville, IN-KY (0021780)	$48.71	$24.32	$80.98	$128.94	$94.13	$253.22
Fairbanks, AK (0021820)	$58.62	$23.19	$106.10	$157.10	$125.55	$274.93
Fargo, ND-MN (0022020)	$54.55	$24.87	$95.24	$144.36	$110.75	$260.97
Fayetteville, NC (0022180)	$42.69	$18.59	$74.58	$117.38	$88.34	$229.45
Flint, MI (0022420)	$55.57	$25.33	$96.02	$145.48	$111.27	$263.44
Florence, SC (0022500)	$50.10	$24.79	$84.41	$132.85	$97.14	$256.14
Fort Collins, CO (0022660)	$56.24	$24.74	$98.49	$148.91	$115.63	$262.44
Fort Wayne, IN (0023060)	$45.07	$20.99	$76.86	$122.05	$91.38	$232.73
Fresno, CA (0023420)	$64.97	$30.91	$111.21	$164.57	$126.25	$288.55
Gainesville, FL (0023540)	$52.67	$26.51	$87.66	$138.09	$102.03	$256.64
Grand Forks, ND-MN (0024220)	$54.09	$24.20	$93.31	$144.09	$109.71	$270.07
Grand Junction, CO (0024300)	$49.03	$22.97	$84.59	$130.57	$97.49	$251.20
Grand Rapids-Wyoming, MI (0024340)	$52.91	$24.79	$89.85	$139.26	$105.18	$255.63
Green Bay, WI (0024580)	$53.29	$23.72	$91.84	$140.54	$107.51	$261.92
Greensboro-High Point, NC (0024660)	$46.95	$19.23	$83.48	$129.40	$99.30	$245.60
Greenville-Anderson-Mauldin, SC (0024860)	$50.18	$24.74	$84.22	$134.18	$98.48	$260.36
Gulfport-Biloxi-Pascagoula, MS (0025060)	$46.16	$22.98	$76.71	$123.77	$89.89	$246.26
Hagerstown-Martinsburg, MD-WV (0025180)	$49.27	$23.09	$84.75	$131.53	$98.09	$248.06
Harrisburg-Carlisle, PA (0025420)	$55.41	$26.94	$92.81	$145.57	$108.71	$269.59
Hartford-West Hartford-East Hartford, CT (0073450)	$62.93	$28.34	$108.64	$162.38	$126.17	$282.96
Hawaii / Kauai nonmetropolitan area (1500001)	$57.13	$24.12	$101.19	$150.54	$117.52	$271.08
Hickory-Lenoir-Morganton, NC (0025860)	$43.60	$18.63	$76.37	$120.49	$90.76	$236.75
Hill Country Region of Texas nonmetropolitan area (4800004)	$49.77	$24.92	$83.26	$131.38	$96.68	$247.71
Hilton Head Island-Bluffton-Beaufort, SC (0025940)	$53.81	$25.62	$91.43	$141.30	$105.91	$264.87
Houston-The Woodlands-Sugar Land, TX (0026420)	$52.86	$25.13	$89.54	$138.90	$103.58	$266.40
Huntington-Ashland, WV-KY-OH (0026580)	$45.89	$23.91	$74.63	$122.80	$87.68	$244.17
Indianapolis-Carmel-Anderson, IN (0026900)	$45.50	$20.90	$78.71	$123.91	$92.26	$241.57
Jackson, MS (0027140)	$46.71	$23.43	$78.10	$124.65	$91.15	$241.27
Jacksonville, FL (0027260)	$54.69	$26.85	$91.61	$141.43	$105.73	$260.26
Kahului-Wailuku-Lahaina, HI (0027980)	$52.80	$23.34	$91.73	$140.12	$107.49	$255.70
Kalamazoo-Portage, MI (0028020)	$54.67	$25.22	$94.07	$144.00	$109.90	$262.16
Kansas City, MO-KS (0028140)	$48.89	$22.71	$83.51	$130.12	$97.31	$252.38
Kennewick-Richland, WA (0028420)	$56.19	$26.09	$97.32	$147.73	$112.56	$263.84
Killeen-Temple, TX (0028660)	$51.31	$25.33	$86.72	$136.22	$100.76	$255.50
Knoxville, TN (0028940)	$47.95	$24.45	$80.08	$128.31	$92.95	$248.95
Lafayette-West Lafayette, IN (0029200)	$43.12	$20.41	$73.61	$117.46	$86.19	$232.86
Lancaster, PA (0029540)	$55.88	$27.09	$94.79	$144.68	$108.63	$265.45
Lansing-East Lansing, MI (0029620)	$56.95	$25.35	$98.94	$148.64	$114.30	$271.74
Laredo, TX (0029700)	$47.83	$24.41	$79.13	$127.44	$92.29	$246.85
Las Cruces, NM (0029740)	$40.24	$17.60	$69.89	$111.76	$83.37	$223.62
Las Vegas-Henderson-Paradise, NV (0029820)	$48.03	$19.30	$86.01	$130.39	$102.20	$238.43
Lexington-Fayette, KY (0030460)	$48.80	$24.39	$81.31	$127.85	$93.32	$251.93
Lima, OH (0030620)	$51.79	$25.28	$87.38	$136.58	$101.44	$257.08
Lincoln, NE (0030700)	$49.91	$23.00	$86.03	$131.75	$98.97	$251.82
Little Rock-North Little Rock-Conway, AR (0030780)	$48.32	$21.17	$84.50	$129.89	$98.93	$241.84
Longview, WA (0031020)	$56.72	$25.85	$96.96	$148.46	$113.47	$268.41
Los Angeles-Long Beach-Anaheim, CA (0031080)	$65.35	$30.86	$110.84	$164.68	$126.55	$291.34
Louisville/Jefferson County, KY-IN (0031140)	$50.95	$22.20	$89.52	$136.78	$105.28	$252.66
Lubbock, TX (0031180)	$49.67	$24.75	$83.50	$131.19	$95.98	$254.32
Madison, WI (0031540)	$48.62	$22.64	$82.62	$130.41	$96.99	$254.09
Manchester, NH (0074950)	$53.70	$24.09	$93.93	$143.13	$109.70	$259.66
Mansfield, OH (0031900)	$47.64	$24.29	$78.62	$125.52	$90.74	$244.39
Maryland nonmetropolitan area (2400006)	$58.33	$28.17	$98.04	$149.80	$113.30	$270.31
Massachusetts nonmetropolitan area (2500006)	$61.39	$29.04	$105.45	$159.01	$122.06	$278.04
McAllen-Edinburg-Mission, TX (0032580)	$48.95	$24.74	$80.97	$128.42	$93.77	$245.04
Medford, OR (0032780)	$55.35	$24.95	$95.79	$147.07	$112.96	$263.96
Memphis, TN-MS-AR (0032820)	$49.46	$21.47	$87.34	$133.77	$102.42	$245.85
Merced, CA (0032900)	$60.64	$29.73	$102.35	$154.82	$116.24	$282.44
Miami-Fort Lauderdale-West Palm Beach, FL (0033100)	$55.91	$27.22	$93.27	$144.41	$108.89	$263.88
Milwaukee-Waukesha-West Allis, WI (0033340)	$51.56	$23.46	$89.81	$136.82	$103.60	$255.18
Minneapolis-St. Paul-Bloomington, MN-WI (0033460)	$52.47	$23.63	$90.55	$140.27	$107.09	$259.53
Missoula, MT (0033540)	$45.23	$19.23	$80.92	$124.96	$95.12	$235.85
Mobile, AL (0033660)	$45.59	$20.49	$79.16	$124.87	$93.65	$241.19
Modesto, CA (0033700)	$67.90	$31.57	$116.36	$170.38	$132.45	$296.26
Montgomery, AL (0033860)	$45.26	$20.42	$78.29	$123.14	$91.99	$239.78
Mount Vernon-Anacortes, WA (0034580)	$57.29	$25.97	$98.63	$149.87	$115.02	$271.91
Mountain North Carolina nonmetropolitan area (3700004)	$41.86	$18.33	$72.70	$115.23	$86.03	$230.10
Napa, CA (0034900)	$78.08	$33.85	$136.93	$196.08	$156.88	$326.14
Naples-Immokalee-Marco Island, FL (0034940)	$52.51	$25.99	$87.89	$137.10	$101.10	$262.24
Nashville-Davidson–Murfreesboro–Franklin, TN (0034980)	$53.49	$25.69	$91.29	$140.74	$105.31	$262.00
Nevada nonmetropolitan area (3200006)	$45.02	$18.42	$79.88	$124.75	$95.85	$241.50
New Bedford, MA (0075550)	$60.97	$28.65	$103.74	$155.98	$119.18	$278.19
New Haven, CT (0075700)	$64.45	$28.64	$112.40	$163.99	$128.68	$285.30
New Orleans-Metairie, LA (0035380)	$49.10	$24.58	$82.09	$130.36	$95.18	$252.46
New York-Newark-Jersey City, NY-NJ-PA (0035620)	$61.66	$28.42	$105.39	$158.43	$122.51	$280.08
North Coast Region of California nonmetropolitan area (0600003)	$62.42	$30.29	$105.02	$158.68	$120.55	$285.40
North Missouri nonmetropolitan area (2900002)	$46.35	$23.30	$77.11	$125.11	$91.20	$241.96
North Northeastern Ohio nonmetropolitan area (3900002)	$49.49	$24.95	$83.70	$130.86	$95.76	$246.91
North Port-Sarasota-Bradenton, FL (0035840)	$53.70	$26.64	$89.43	$139.96	$104.24	$258.18
North Texas Region of Texas nonmetropolitan area (4800002)	$51.64	$25.42	$86.00	$135.67	$101.13	$252.79
North Valley-Northern Mountains Region of California nonmetropolitan area (0600007)	$63.67	$30.65	$107.83	$162.54	$123.78	$285.86
Northeast Coastal North Carolina nonmetropolitan area (3700002)	$43.19	$18.83	$75.96	$119.08	$90.13	$228.95
Northeast Lower Peninsula of Michigan nonmetropolitan area (2600002)	$52.02	$24.42	$89.10	$137.40	$103.16	$256.26
Northeast Mississippi nonmetropolitan area (2800001)	$44.01	$22.71	$72.81	$118.41	$84.34	$236.26
Northeast Oklahoma nonmetropolitan area (4000001)	$45.34	$22.61	$75.87	$121.74	$88.25	$240.25
Northeast South Carolina nonmetropolitan area (4500006)	$51.93	$25.25	$87.10	$137.19	$101.72	$259.05
Northeastern Wisconsin nonmetropolitan area (5500002)	$51.58	$23.42	$89.00	$138.96	$105.25	$261.79
Northern Indiana nonmetropolitan area (1800001)	$43.41	$20.24	$73.63	$118.72	$87.07	$240.98
Northern New Mexico nonmetropolitan area (3500006)	$41.79	$17.71	$73.53	$116.41	$87.48	$235.08
Northern Pennsylvania nonmetropolitan area (4200002)	$53.57	$26.50	$89.29	$139.64	$103.79	$264.60
Northern Vermont nonmetropolitan area (5000002)	$54.98	$27.60	$91.89	$141.55	$105.68	$258.03
Northern West Virginia nonmetropolitan area (5400002)	$46.33	$22.40	$78.43	$125.15	$91.48	$241.59
Northwest Colorado nonmetropolitan area (0800003)	$52.65	$23.37	$91.38	$140.43	$107.07	$266.33
Northwest Illinois nonmetropolitan area (1700001)	$43.28	$20.52	$74.35	$118.42	$86.75	$231.33
Northwest Lower Peninsula of Michigan nonmetropolitan area (2600003)	$51.55	$24.30	$87.74	$136.51	$102.25	$256.58
Northwest Minnesota nonmetropolitan area (2700001)	$52.17	$24.14	$90.11	$138.87	$105.08	$255.46
Northwest Mississippi nonmetropolitan area (2800002)	$46.10	$23.25	$76.52	$122.93	$89.18	$239.66
Northwestern Idaho nonmetropolitan area (1600006)	$44.84	$20.86	$77.15	$121.71	$90.55	$235.07
Ogden-Clearfield, UT (0036260)	$45.33	$20.32	$78.76	$124.43	$92.90	$244.41
Oklahoma City, OK (0036420)	$49.46	$23.44	$84.75	$132.40	$98.32	$253.29
Olympia-Tumwater, WA (0036500)	$56.51	$26.11	$96.47	$147.36	$112.97	$265.69
Omaha-Council Bluffs, NE-IA (0036540)	$53.80	$23.47	$94.43	$142.65	$109.82	$263.00
Orlando-Kissimmee-Sanford, FL (0036740)	$53.93	$26.54	$90.91	$139.79	$103.78	$263.42
Oxnard-Thousand Oaks-Ventura, CA (0037100)	$65.10	$31.14	$110.26	$164.28	$126.42	$285.82
Palm Bay-Melbourne-Titusville, FL (0037340)	$54.95	$26.81	$92.72	$143.32	$107.14	$264.94
Pensacola-Ferry Pass-Brent, FL (0037860)	$52.46	$26.44	$87.73	$138.03	$101.78	$258.70
Peoria, IL (0037900)	$43.69	$20.55	$74.80	$119.13	$87.70	$234.17
Philadelphia-Camden-Wilmington, PA-NJ-DE-MD (0037980)	$60.89	$28.72	$103.28	$155.58	$119.14	$277.09
Phoenix-Mesa-Scottsdale, AZ (0038060)	$47.00	$21.83	$80.88	$127.56	$94.66	$251.02
Piedmont North Carolina nonmetropolitan area (3700003)	$43.31	$18.84	$75.97	$118.43	$89.48	$228.62
Pittsburgh, PA (0038300)	$55.23	$26.68	$93.06	$143.39	$107.28	$266.14
Port St. Lucie, FL (0038940)	$53.49	$26.28	$89.50	$139.66	$103.49	$267.85
Portland-South Portland, ME (0076750)	$62.00	$29.22	$106.14	$159.89	$122.97	$277.96
Portland-Vancouver-Hillsboro, OR-WA (0038900)	$62.11	$27.16	$108.48	$160.34	$125.79	$280.95
Providence-Warwick, RI-MA (0077200)	$65.46	$29.35	$115.09	$168.40	$130.69	$297.89
Provo-Orem, UT (0039340)	$46.69	$20.68	$80.80	$126.67	$95.65	$241.56
Raleigh, NC (0039580)	$48.10	$19.57	$85.93	$130.41	$101.25	$245.27
Reading, PA (0039740)	$52.87	$26.35	$87.83	$137.74	$101.74	$259.06
Reno, NV (0039900)	$50.60	$19.77	$92.85	$138.76	$109.44	$249.71
Richmond, VA (0040060)	$51.86	$21.77	$92.05	$139.30	$108.41	$254.00
Riverside-San Bernardino-Ontario, CA (0040140)	$66.70	$31.23	$114.98	$167.24	$129.41	$293.30
Roanoke, VA (0040220)	$47.16	$20.68	$82.21	$127.51	$96.33	$244.50
Rochester, MN (0040340)	$57.12	$25.29	$99.46	$149.85	$116.47	$268.44
Rochester, NY (0040380)	$44.36	$18.39	$78.84	$122.34	$93.41	$236.87
Rockford, IL (0040420)	$44.21	$20.52	$75.37	$120.43	$89.20	$238.45
Sacramento–Roseville–Arden-Arcade, CA (0040900)	$71.45	$32.74	$123.16	$179.34	$141.39	$301.61
Salem, OR (0041420)	$53.20	$24.33	$92.10	$141.44	$107.52	$261.98
Salinas, CA (0041500)	$64.18	$30.93	$109.00	$162.47	$124.27	$285.97
Salisbury, MD-DE (0041540)	$61.13	$30.15	$102.38	$155.73	$117.63	$278.87
Salt Lake City, UT (0041620)	$49.18	$20.95	$86.49	$134.03	$102.39	$256.64
San Antonio-New Braunfels, TX (0041700)	$51.17	$25.13	$85.93	$134.83	$99.88	$256.20
San Diego-Carlsbad, CA (0041740)	$64.62	$30.90	$109.74	$164.96	$126.79	$287.19
San Francisco-Oakland-Hayward, CA (0041860)	$74.31	$33.42	$128.06	$186.37	$148.22	$307.62
San Jose-Sunnyvale-Santa Clara, CA (0041940)	$75.02	$33.32	$130.80	$186.48	$148.28	$311.50
San Juan-Carolina-Caguas, PR (0041980)	$45.52	$25.26	$72.77	$120.25	$83.44	$246.71
San Luis Obispo-Paso Robles-Arroyo Grande, CA (0042020)	$70.93	$32.71	$122.62	$178.49	$140.17	$297.92
Santa Cruz-Watsonville, CA (0042100)	$67.11	$31.46	$114.85	$169.84	$131.48	$295.95
Santa Maria-Santa Barbara, CA (0042200)	$66.42	$31.19	$114.17	$167.47	$129.27	$293.50
Santa Rosa, CA (0042220)	$70.28	$32.04	$121.60	$177.87	$139.03	$307.08
Scranton–Wilkes-Barre–Hazleton, PA (0042540)	$53.39	$26.63	$88.64	$137.94	$102.86	$256.65
Seattle-Tacoma-Bellevue, WA (0042660)	$59.46	$26.49	$102.54	$154.40	$119.81	$276.24
Sioux City, IA-NE-SD (0043580)	$44.81	$18.83	$79.91	$121.80	$93.81	$227.43
South Central Kentucky nonmetropolitan area (2100002)	$46.25	$23.93	$75.61	$123.58	$89.06	$245.59
South Central Wisconsin nonmetropolitan area (5500003)	$50.83	$23.11	$87.09	$135.09	$101.96	$257.35
South Illinois nonmetropolitan area (1700004)	$42.16	$20.19	$71.44	$115.06	$83.88	$230.78
South Nebraska nonmetropolitan area (3100006)	$45.88	$22.24	$77.55	$123.43	$90.50	$241.42
Southeast Coastal North Carolina nonmetropolitan area (3700001)	$42.99	$18.74	$74.78	$117.78	$88.99	$229.19
Southeast Minnesota nonmetropolitan area (2700004)	$49.68	$23.36	$84.83	$132.71	$98.58	$254.37
Southeast Mississippi nonmetropolitan area (2800003)	$42.95	$22.43	$70.32	$116.43	$82.47	$235.99
Southeast Missouri nonmetropolitan area (2900003)	$46.72	$23.12	$78.72	$126.05	$91.33	$249.34
Southeast Oklahoma nonmetropolitan area (4000004)	$45.76	$22.54	$76.81	$122.52	$89.32	$243.07
Southern Indiana nonmetropolitan area (1800003)	$42.51	$20.33	$72.64	$116.05	$84.70	$228.60
Southern Ohio nonmetropolitan area (3900004)	$51.81	$25.10	$87.10	$136.95	$101.41	$258.91
Southern West Virginia nonmetropolitan area (5400001)	$42.52	$21.50	$70.92	$116.64	$83.09	$236.85
Southwest Colorado nonmetropolitan area (0800002)	$53.17	$23.83	$92.61	$142.67	$108.89	$263.74
Southwest Iowa nonmetropolitan area (1900003)	$52.38	$23.29	$91.17	$139.65	$106.71	$257.29
Southwest Maine nonmetropolitan area (2300002)	$58.12	$28.06	$98.79	$150.12	$113.04	$269.90
Southwest Mississippi nonmetropolitan area (2800004)	$43.86	$22.59	$72.58	$116.16	$82.95	$235.57
Southwest Missouri nonmetropolitan area (2900004)	$46.75	$23.22	$77.51	$124.14	$90.96	$245.40
Southwest Montana nonmetropolitan area (3000003)	$45.41	$19.37	$80.73	$124.68	$95.37	$231.80
Southwest New York nonmetropolitan area (3600004)	$45.14	$18.64	$80.79	$125.09	$95.68	$237.66
Spokane-Spokane Valley, WA (0044060)	$55.47	$25.85	$95.77	$143.64	$109.28	$259.17
Springfield, IL (0044100)	$48.14	$21.64	$83.85	$129.96	$98.68	$243.48
Springfield, MA-CT (0078100)	$55.99	$26.56	$95.97	$145.85	$109.83	$270.64
Springfield, MO (0044180)	$47.98	$23.72	$80.99	$128.03	$94.04	$243.67
St. Cloud, MN (0041060)	$51.97	$23.91	$87.87	$137.31	$103.99	$257.62
St. Louis, MO-IL (0041180)	$45.76	$20.89	$79.37	$125.12	$93.02	$240.39
Stockton-Lodi, CA (0044700)	$64.97	$30.99	$109.62	$163.48	$125.95	$286.07
Syracuse, NY (0045060)	$45.64	$18.87	$80.82	$124.36	$95.98	$232.34
Tallahassee, FL (0045220)	$51.51	$25.94	$85.78	$135.95	$98.98	$260.06
Tampa-St. Petersburg-Clearwater, FL (0045300)	$54.85	$26.93	$92.11	$142.63	$106.88	$263.81
Terre Haute, IN (0045460)	$41.68	$20.21	$70.67	$113.60	$82.50	$224.49
Toledo, OH (0045780)	$52.39	$25.35	$88.85	$138.56	$102.86	$260.89
Trenton, NJ (0045940)	$48.58	$22.81	$82.65	$129.89	$97.05	$250.32
Tucson, AZ (0046060)	$44.89	$21.48	$75.57	$120.82	$88.67	$240.10
Tulsa, OK (0046140)	$48.30	$23.11	$81.35	$128.95	$95.50	$250.49
Tyler, TX (0046340)	$49.93	$24.68	$84.06	$132.28	$96.74	$257.24
Upper Peninsula of Michigan nonmetropolitan area (2600001)	$50.28	$24.00	$84.98	$132.22	$98.29	$252.83
Urban Honolulu, HI (0046520)	$53.02	$23.22	$92.88	$140.12	$107.21	$260.80
Utica-Rome, NY (0046540)	$43.92	$18.52	$77.27	$120.24	$92.10	$228.76
Vallejo-Fairfield, CA (0046700)	$71.68	$32.48	$121.88	$180.23	$142.08	$305.14
Virginia Beach-Norfolk-Newport News, VA-NC (0047260)	$46.37	$19.40	$82.85	$127.18	$97.57	$237.36
Visalia-Porterville, CA (0047300)	$65.52	$31.15	$111.21	$165.57	$127.32	$287.67
Waco, TX (0047380)	$52.82	$25.75	$89.04	$139.07	$104.07	$256.27
Washington-Arlington-Alexandria, DC-VA-MD-WV (0047900)	$54.59	$24.12	$94.70	$144.52	$111.15	$264.86
Waterloo-Cedar Falls, IA (0047940)	$55.00	$23.57	$97.10	$146.54	$113.28	$268.38
Watertown-Fort Drum, NY (0048060)	$41.84	$17.99	$73.57	$115.84	$87.38	$226.23
West Central Illinois nonmetropolitan area (1700002)	$45.41	$20.69	$78.48	$124.08	$92.12	$243.41
West Central-Southwest New Hampshire nonmetropolitan area (3300006)	$47.90	$22.62	$81.64	$128.52	$95.44	$250.35
West Kentucky nonmetropolitan area (2100001)	$50.03	$24.75	$84.53	$133.04	$97.49	$255.63
West Montana nonmetropolitan area (3000004)	$45.54	$18.97	$80.61	$124.70	$95.09	$241.07
West Texas Region of Texas nonmetropolitan area (4800001)	$48.35	$24.41	$81.05	$128.61	$93.17	$250.91
Western Washington nonmetropolitan area (5300006)	$56.33	$26.01	$97.27	$147.23	$112.32	$263.56
Western Wisconsin nonmetropolitan area (5500004)	$52.19	$23.58	$90.41	$139.35	$105.97	$259.77
Western Wyoming nonmetropolitan area (5600006)	$46.41	$19.73	$81.58	$127.36	$97.19	$243.81
Wichita Falls, TX (0048660)	$48.96	$24.55	$81.12	$129.37	$94.75	$252.08
Wichita, KS (0048620)	$47.99	$22.79	$81.81	$128.74	$95.53	$244.26
Wilmington, NC (0048900)	$44.13	$18.74	$77.54	$121.05	$91.93	$236.86
Winston-Salem, NC (0049180)	$45.76	$19.25	$81.37	$125.60	$96.38	$238.51
Worcester, MA-CT (0079600)	$58.18	$27.11	$100.12	$150.66	$114.61	$271.53
Yakima, WA (0049420)	$57.05	$26.04	$97.17	$148.65	$113.83	$268.66
York-Hanover, PA (0049620)	$53.68	$26.41	$90.17	$140.43	$104.08	$265.08
Youngstown-Warren-Boardman, OH-PA (0049660)	$54.65	$26.64	$91.71	$141.79	$105.68	$267.72
Yuma, AZ (0049740)	$42.73	$21.05	$71.73	$116.00	$83.69	$235.22

The minimum fee-for-service (FFS) payment rate was computed for a 30-min CHW visit (Medicaid billing code 98960), and the minimum capitated payment rate per member per month (PMPM), after considering the wages and overhead costs for CHW programs

**Table 6:** Sensitivity analysis when we swapped out the expenditures for an RN care manager supervisor (1 for each 8 CHWs) for an MSW supervisor (1 for each six CHWs)

State	Minimum fee-for-service (FFS) payment per visit	95% CI (FFS lower)	95% CI (FFS upper)	Minimum capitated payment per month per patient (PMPM)	95% CI (PMPM lower)	95% CI (PMPM upper)
Alabama	$46.88	$24.95	$76.28	$124.17	$88.38	$244.57
Alaska	$55.85	$22.83	$99.32	$148.05	$117.38	$259.50
Arizona	$51.38	$25.08	$87.15	$135.42	$100.47	$255.27
Arkansas	$44.69	$21.57	$75.45	$120.18	$87.94	$237.25
California	$66.35	$31.73	$112.41	$166.56	$129.31	$283.75
Colorado	$53.22	$23.97	$92.34	$141.23	$107.80	$258.23
Connecticut	$62.07	$28.41	$107.07	$160.70	$124.98	$277.75
Delaware	$53.61	$24.74	$92.39	$142.00	$106.58	$272.07
District of Columbia	$69.55	$32.18	$119.53	$174.37	$136.33	$297.45
Florida	$53.61	$26.36	$89.96	$141.10	$104.83	$268.12
Georgia	$55.84	$27.88	$93.09	$144.64	$107.58	$266.53
Hawaii	$51.19	$23.03	$88.32	$135.48	$102.61	$252.98
Idaho	$48.59	$20.96	$84.98	$131.29	$100.30	$249.05
Illinois	$58.51	$29.25	$96.40	$149.81	$112.13	$276.58
Indiana	$47.07	$22.52	$79.71	$127.16	$94.12	$247.43
Iowa	$45.71	$21.06	$78.71	$123.34	$91.98	$235.60
Kansas	$48.37	$22.75	$82.36	$130.73	$97.10	$253.41
Kentucky	$53.55	$25.67	$90.27	$139.34	$104.72	$259.30
Louisiana	$51.38	$25.31	$85.32	$133.67	$99.80	$250.38
Maine	$47.16	$20.32	$83.00	$128.86	$98.10	$243.82
Maryland	$56.79	$27.71	$95.23	$147.74	$111.15	$269.53
Massachusetts	$61.76	$28.62	$105.90	$156.86	$120.25	$282.00
Michigan	$53.20	$24.71	$91.27	$141.20	$106.68	$261.31
Minnesota	$52.16	$24.15	$88.69	$139.30	$105.60	$256.17
Mississippi	$44.44	$22.34	$73.33	$118.54	$85.80	$234.98
Missouri	$49.00	$23.73	$82.63	$130.40	$96.11	$250.04
Montana	$44.93	$19.04	$79.75	$122.71	$93.11	$234.06
Nebraska	$49.10	$23.15	$82.73	$130.50	$98.05	$245.22
Nevada	$56.48	$26.53	$96.50	$147.07	$112.26	$265.27
New Hampshire	$53.73	$24.77	$91.62	$142.26	$107.86	$263.58
New Jersey	$64.54	$31.45	$108.99	$162.34	$123.58	$285.11
New Mexico	$56.54	$25.41	$97.28	$148.63	$114.38	$271.28
New York	$65.86	$31.42	$111.12	$165.77	$127.98	$285.73
North Carolina	$54.28	$26.80	$91.33	$140.97	$105.19	$261.36
North Dakota	$49.95	$19.64	$90.45	$136.73	$107.20	$246.51
Ohio	$51.77	$25.24	$87.38	$135.26	$100.23	$255.07
Oklahoma	$47.72	$22.87	$81.23	$127.68	$93.85	$252.15
Oregon	$55.23	$24.53	$96.61	$145.30	$112.03	$266.40
Pennsylvania	$56.45	$27.69	$95.28	$145.66	$109.93	$259.50
Puerto Rico	$46.62	$25.66	$74.56	$122.16	$85.71	$244.09
Rhode Island	$62.20	$28.06	$106.46	$160.20	$124.28	$281.74
South Carolina	$51.21	$25.00	$86.45	$135.85	$100.33	$261.69
South Dakota	$40.83	$17.90	$72.37	$113.91	$85.32	$222.46
Tennessee	$50.75	$25.28	$84.76	$134.11	$99.34	$250.39
Texas	$50.44	$25.05	$85.17	$132.61	$97.48	$250.69
Utah	$48.64	$21.14	$85.34	$131.83	$100.63	$245.54
Vermont	$49.21	$21.34	$86.09	$133.12	$101.91	$246.40
Virginia	$58.94	$27.66	$100.24	$152.22	$115.02	$284.52
Washington	$56.56	$25.88	$97.34	$147.91	$113.18	$269.31
West Virginia	$43.97	$21.78	$73.82	$118.71	$85.82	$238.44
Wisconsin	$51.10	$23.52	$88.07	$136.24	$103.19	$252.41
Wyoming	$46.64	$19.74	$81.95	$127.13	$97.20	$240.82

The minimum fee-for-service (FFS) payment rate was computed for a 30-min CHW visit (Medicaid billing code 98960), and the minimum capitated payment rate per member per month (PMPM), after considering the wages and overhead costs for CHW programs

**Table 7:** Sensitivity analysis when we reduced the typical cross-sectional patient panel size per CHW from 65 to 15

State	Minimum fee-for-service (FFS) payment per visit	95% CI (FFS Lower)	95% CI (FFS Upper)	Minimum capitated payment per month per patient (PMPM)	95% CI (PMPM Lower)	95% CI (PMPM Upper)
Alabama	$204.10	$108.46	$332.41	$539.33	$385.71	$1057.29
Alaska	$246.52	$98.32	$441.61	$651.82	$517.27	$1155.79
Arizona	$225.38	$109.89	$377.74	$591.80	$441.18	$1103.14
Arkansas	$193.01	$92.99	$326.26	$520.09	$381.42	$1041.39
California	$293.84	$136.41	$505.87	$741.78	$575.60	$1294.11
Colorado	$234.48	$103.74	$412.06	$619.97	$475.02	$1140.27
Connecticut	$270.05	$122.07	$463.97	$697.15	$542.53	$1217.23
Delaware	$236.99	$109.37	$404.86	$618.11	$467.57	$1155.09
District of Columbia	$307.19	$139.01	$521.08	$765.18	$603.46	$1322.23
Florida	$235.95	$116.57	$399.36	$615.77	$460.20	$1129.48
Georgia	$245.70	$120.94	$411.67	$635.27	$475.50	$1184.34
Hawaii	$231.36	$101.79	$404.95	$616.11	$472.55	$1124.37
Idaho	$207.87	$90.74	$363.83	$568.79	$432.51	$1074.58
Illinois	$255.06	$127.14	$426.36	$653.55	$485.46	$1202.85
Indiana	$205.18	$97.63	$350.13	$552.63	$408.85	$1071.72
Iowa	$196.78	$90.52	$339.86	$535.77	$399.75	$1034.89
Kansas	$212.12	$99.41	$362.09	$561.99	$419.73	$1062.45
Kentucky	$233.26	$110.50	$397.37	$609.09	$456.65	$1152.06
Louisiana	$222.39	$109.59	$375.57	$588.90	$436.54	$1108.73
Maine	$206.66	$87.92	$363.00	$559.35	$426.57	$1070.46
Maryland	$249.73	$119.64	$425.97	$646.19	$485.51	$1196.00
Massachusetts	$273.35	$125.62	$471.47	$697.71	$536.73	$1234.05
Michigan	$233.18	$107.29	$398.88	$609.83	$461.41	$1130.18
Minnesota	$230.45	$103.91	$398.36	$614.90	$465.18	$1161.64
Mississippi	$189.37	$96.50	$311.74	$512.59	$368.33	$1023.75
Missouri	$214.33	$104.00	$360.10	$563.94	$419.47	$1069.12
Montana	$197.30	$83.37	$345.41	$537.90	$414.27	$1022.71
Nebraska	$214.28	$99.97	$361.79	$567.19	$426.66	$1077.70
Nevada	$252.46	$115.61	$434.37	$651.13	$500.89	$1179.10
New Hampshire	$231.66	$107.73	$402.35	$616.03	$465.49	$1128.23
New Jersey	$280.93	$136.02	$473.94	$710.41	$541.45	$1254.63
New Mexico	$245.70	$110.11	$426.57	$644.84	$496.95	$1174.59
New York	$289.29	$135.50	$494.13	$733.20	$567.97	$1280.80
North Carolina	$236.25	$115.87	$399.88	$614.34	$457.86	$1140.36
North Dakota	$215.93	$83.29	$389.26	$592.54	$465.57	$1102.27
Ohio	$225.07	$108.98	$376.91	$590.29	$439.49	$1122.07
Oklahoma	$206.18	$99.10	$349.83	$559.82	$410.45	$1097.29
Oregon	$247.95	$108.07	$431.04	$652.82	$504.96	$1190.24
Pennsylvania	$243.06	$117.69	$408.76	$626.47	$471.03	$1133.73
Puerto Rico	$193.27	$108.38	$306.28	$514.11	$353.64	$1057.38
Rhode Island	$277.42	$122.63	$484.81	$720.68	$561.21	$1264.38
South Carolina	$220.35	$108.46	$369.33	$579.37	$427.74	$1109.16
South Dakota	$175.24	$76.48	$308.06	$492.05	$366.51	$992.46
Tennessee	$223.60	$109.03	$374.83	$586.56	$436.84	$1112.24
Texas	$221.48	$108.94	$375.61	$587.34	$433.25	$1127.45
Utah	$210.86	$92.30	$369.20	$569.88	$435.85	$1054.47
Vermont	$212.16	$91.56	$372.58	$576.51	$441.57	$1086.54
Virginia	$255.88	$122.89	$433.98	$659.14	$501.41	$1183.78
Washington	$251.51	$113.36	$438.88	$657.54	$504.66	$1194.74
West Virginia	$188.67	$94.47	$314.82	$508.04	$367.34	$1019.37
Wisconsin	$223.21	$101.70	$384.19	$596.14	$453.18	$1112.37
Wyoming	$203.28	$85.84	$357.63	$555.88	$425.88	$1053.22

The minimum fee-for-service (FFS) payment rate was computed for a 30-min CHW visit (Medicaid billing code 98960), and the minimum capitated payment rate per member per month (PMPM), after considering the wages and overhead costs for CHW programs

**Table 8:** Sensitivity analysis when we increased the typical cross-sectional patient panel size per CHW from 65 to 100

State	Minimum fee-for-service (FFS) payment per visit	95% CI (FFS lower)	95% CI (FFS upper)	Minimum capitated payment per month per patient (PMPM)	95% CI (PMPM lower)	95% CI (PMPM upper)
Alabama	$30.62	$16.27	$49.86	$80.90	$57.86	$158.59
Alaska	$36.98	$14.75	$66.24	$97.77	$77.59	$173.37
Arizona	$33.81	$16.48	$56.66	$88.77	$66.18	$165.47
Arkansas	$28.95	$13.95	$48.94	$78.01	$57.21	$156.21
California	$44.08	$20.46	$75.88	$111.27	$86.34	$194.12
Colorado	$35.17	$15.56	$61.81	$93.00	$71.25	$171.04
Connecticut	$40.51	$18.31	$69.60	$104.57	$81.38	$182.59
Delaware	$35.55	$16.41	$60.73	$92.72	$70.14	$173.26
District of Columbia	$46.08	$20.85	$78.16	$114.78	$90.52	$198.33
Florida	$35.39	$17.49	$59.90	$92.37	$69.03	$169.42
Georgia	$36.86	$18.14	$61.75	$95.29	$71.32	$177.65
Hawaii	$34.70	$15.27	$60.74	$92.42	$70.88	$168.66
Idaho	$31.18	$13.61	$54.57	$85.32	$64.88	$161.19
Illinois	$38.26	$19.07	$63.95	$98.03	$72.82	$180.43
Indiana	$30.78	$14.64	$52.52	$82.89	$61.33	$160.76
Iowa	$29.52	$13.58	$50.98	$80.37	$59.96	$155.23
Kansas	$31.82	$14.91	$54.31	$84.30	$62.96	$159.37
Kentucky	$34.99	$16.58	$59.61	$91.36	$68.50	$172.81
Louisiana	$33.36	$16.44	$56.34	$88.34	$65.48	$166.31
Maine	$31.00	$13.19	$54.45	$83.90	$63.99	$160.57
Maryland	$37.46	$17.95	$63.90	$96.93	$72.83	$179.40
Massachusetts	$41.00	$18.84	$70.72	$104.66	$80.51	$185.11
Michigan	$34.98	$16.09	$59.83	$91.47	$69.21	$169.53
Minnesota	$34.57	$15.59	$59.75	$92.24	$69.78	$174.25
Mississippi	$28.41	$14.48	$46.76	$76.89	$55.25	$153.56
Missouri	$32.15	$15.60	$54.02	$84.59	$62.92	$160.37
Montana	$29.59	$12.51	$51.81	$80.68	$62.14	$153.41
Nebraska	$32.14	$15.00	$54.27	$85.08	$64.00	$161.66
Nevada	$37.87	$17.34	$65.16	$97.67	$75.13	$176.87
New Hampshire	$34.75	$16.16	$60.35	$92.40	$69.82	$169.23
New Jersey	$42.14	$20.40	$71.09	$106.56	$81.22	$188.19
New Mexico	$36.86	$16.52	$63.99	$96.73	$74.54	$176.19
New York	$43.39	$20.33	$74.12	$109.98	$85.20	$192.12
North Carolina	$35.44	$17.38	$59.98	$92.15	$68.68	$171.05
North Dakota	$32.39	$12.49	$58.39	$88.88	$69.84	$165.34
Ohio	$33.76	$16.35	$56.54	$88.54	$65.92	$168.31
Oklahoma	$30.93	$14.87	$52.47	$83.97	$61.57	$164.59
Oregon	$37.19	$16.21	$64.66	$97.92	$75.74	$178.54
Pennsylvania	$36.46	$17.65	$61.31	$93.97	$70.66	$170.06
Puerto Rico	$28.99	$16.26	$45.94	$77.12	$53.05	$158.61
Rhode Island	$41.61	$18.40	$72.72	$108.10	$84.18	$189.66
South Carolina	$33.05	$16.27	$55.40	$86.91	$64.16	$166.37
South Dakota	$26.29	$11.47	$46.21	$73.81	$54.98	$148.87
Tennessee	$33.54	$16.35	$56.23	$87.98	$65.53	$166.84
Texas	$33.22	$16.34	$56.34	$88.10	$64.99	$169.12
Utah	$31.63	$13.85	$55.38	$85.48	$65.38	$158.17
Vermont	$31.82	$13.73	$55.89	$86.48	$66.24	$162.98
Virginia	$38.38	$18.43	$65.10	$98.87	$75.21	$177.57
Washington	$37.73	$17.00	$65.83	$98.63	$75.70	$179.21
West Virginia	$28.30	$14.17	$47.22	$76.21	$55.10	$152.91
Wisconsin	$33.48	$15.26	$57.63	$89.42	$67.98	$166.86
Wyoming	$30.49	$12.88	$53.64	$83.38	$63.88	$157.98

The minimum fee-for-service (FFS) payment rate was computed for a 30-min CHW visit (Medicaid billing code 98960), and the minimum capitated payment rate per member per month (PMPM), after considering the wages and overhead costs for CHW programs
